# One to host them all: genomics of the diverse bacterial endosymbionts of the spider *Oedothorax gibbosus*

**DOI:** 10.1099/mgen.0.000943

**Published:** 2023-02-09

**Authors:** Tamara Halter, Stephan Köstlbacher, Thomas Rattei, Frederik Hendrickx, Alejandro Manzano-Marín, Matthias Horn

**Affiliations:** ^1^​ Centre for Microbiology and Environmental Systems Science, University of Vienna. Djerassiplatz 1, 1030 Vienna, Austria; ^2^​ Doctoral School in Microbiology and Environmental Science, University of Vienna. Universitätsring 1, 1010 Vienna, Austria; ^3^​ Current address: Laboratory of Microbiology, Wageningen University and Research, Stippeneng 4, 6700 EH Wageningen, The Netherlands; ^4^​ OD Taxonomy and Phylogeny, Royal Belgian Institute of Natural Sciences. Rue Vautier/Vautierstraat 29,, 1000 Brussels, Belgium

**Keywords:** spider endosymbionts, comparative genomics, mobile elements, host–symbiont interaction

## Abstract

Bacterial endosymbionts of the groups *Wolbachia *, *Cardinium* and *Rickettsiaceae* are well known for their diverse effects on their arthropod hosts, ranging from mutualistic relationships to reproductive phenotypes. Here, we analysed a unique system in which the dwarf spider *Oedothorax gibbosus* is co-infected with up to five different endosymbionts affiliated with *Wolbachia *, ‘*Candidatus* Tisiphia’ (formerly Torix group *Rickettsia *), *Cardinium* and *Rhabdochlamydia *. Using short-read genome sequencing data, we show that the endosymbionts are heterogeneously distributed among *O. gibbosus* populations and are frequently found co-infecting spider individuals. To study this intricate host–endosymbiont system on a genome-resolved level, we used long-read sequencing to reconstruct closed genomes of the *Wolbachia *, ‘*Ca*. Tisiphia’ and *Cardinium* endosymbionts. We provide insights into the ecology and evolution of the endosymbionts and shed light on the interactions with their spider host. We detected high quantities of transposable elements in all endosymbiont genomes and provide evidence that ancestors of the *Cardinium *, ‘*Ca*. Tisiphia’ and *Wolbachia* endosymbionts have co-infected the same hosts in the past. Our findings contribute to broadening our knowledge about endosymbionts infecting one of the largest animal phyla on Earth and show the usefulness of transposable elements as an evolutionary ‘contact-tracing’ tool.

## Data Summary

All supporting data, code and protocols have been provided within the article or through supplementary data files. Seven supplementary figures and eight supplementary tables are available with the online version of this article. Sequencing data used in this study was generated and previously published by Hendrickx *et al*. in 2021. Genome assemblies generated in this study have been deposited under the project PRJEB52003 at DDBJ/ENA/GenBank. The MAG of *R. oedothoracis* OV001 was deposited at DDBJ/ENA/GenBank under the sample SAMN28026840. The genome of ‘*Candidatus* Rhabdochlamydia oedothoracis W744×776’ was previously published by Halter *et al*. n 2022 and is available at DDBJ/ENA/GenBank (accession: CP075587-CP075588). The collection of genomes and proteomes, all files for phylogenetic analyses including gene alignments, concatenated alignments and tree files, and original output files of the HGT and SNP predictions used in this study are available at Zenodo (https://doi.org/10.5281/zenodo.6362846).

**Impact Statement**

Studies investigating the symbionts of spiders have focused mostly on amplicon sequencing to describe the community composition. However, to gain insights into functions and evolution, whole genome information is required. Here, we were able to reconstruct the complete genomes of four different endosymbionts belonging to the important groups *Wolbachia *, *Cardinium* and *Rickettsiaceae* infecting a single host – the spider *Oedothorax gibbosus*. In our analysis we could show that co-infections with multiple endosymbionts happen frequently in the spider, which was previously assumed to be a rare phenomenon. Further, we conducted an in-depth analysis of the genomes and provided insights into the interactions of the endosymbionts with their host. We also identified a high abundance of transposable elements in the genomes of all endosymbionts and used them as acontact-tracing tool to show that the endosymbionts have already co-existed in the past.

## Introduction

Arthropods are the largest and most diverse animal phylum on the planet [1], and they are frequently infected with endosymbiotic bacteria. Endosymbionts can have a variety of influences on their arthropod hosts including the manipulation of sexual reproduction, nutrition or resistance to pathogens [2]. Maternally transmitted endosymbionts belonging to the groups *Wolbachia *, *Cardinium* and *Rickettsiaceae* are of particular interest because of the ability of some strains to induce reproductive phenotypes in their animal hosts [3–7]. These bacteria are also the most prevalent arthropod endosymbionts infecting around a half, a quarter and an eighth of terrestrial arthropod species, respectively [2].

The genus *Wolbachia* (* Alphaproteobacteria *) includes primarily maternally transmitted endosymbionts of arthropods and nematodes, organized into at least 17 phylogenetic supergroups, where supergroup A and B are the most-well studied [6]. *Wolbachia* endosymbionts are most famous for the array of reproductive manipulations they can cause in their hosts, ranging from cytoplasmic incompatibility to male killing, parthenogenesis and feminization [6]. However, they can also act as obligate or facultative mutualistic symbionts and have been reported to increase resistance against viruses in some host species [6]. Like *Wolbachia *, members of the genus *Cardinium* (* Bacteroidetes *) are endosymbionts of arthropods and nematodes [8]. Apart from causing reproductive manipulations [4, 7, 9, 10] *Cardinium* can be involved in the provision of nutrients [11] and in shaping its host’s microbiome [12, 13]. The family *Rickettsiaceae* (* Alphaproteobacteria *) includes the well-known arthropod-transmitted vertebrate and human pathogens of the genus *Rickettsia* but also invertebrate-restricted endosymbionts that are not transferred to vertebrate hosts and were recently placed in a separate genus ‘*Ca*. Tisiphia’ (formerly Torix group *Rickettsia *) [14, 15]. Like *Wolbachia* and *Cardinium *, ‘*Ca*. Tisiphia’ include not only reproductive parasites that manipulate their host’s reproduction [3, 5, 16, 17] but also mutualists that can increase resistance to pathogenic fungi, bacteria and viruses [18–20].

Based on rRNA gene sequence analysis, the microbiome of the spider *Oedothorax gibbosus* was previously shown to be dominated by endosymbionts belonging to the above-mentioned genera and a less prevalent symbiont belonging to *Rhabdochlamydia* [21]. The genus *Rhabdochlamydia* belongs to the phylum *Chlamydiae *, which consists of obligate intracellular bacteria [22] including major human and vertebrate pathogens, such as *Chlamydia trachomatis,* as well as symbionts of amoeba, and diverse environmental lineages with unknown hosts [22]. Known *Rhabdochlamydia* species are primarily endosymbionts of arthropods [21, 23–25], but their influence on the host remains to be investigated. However, there are indications that they have pathogenic rather than mutualistic effects [24, 26, 27]. Apart from harbouring mainly endosymbionts, *O. gibbosus* was previously shown to be co-infected with multiple endosymbionts at the same time [21], which has only been described for a few other hosts previously.

Like for *O. gibbosus,* most previous studies looking at spider endosymbionts have so far focused on describing the microbiome composition [21, 28–32]. However, whole genome information is needed to get deeper insights into host–symbiont interactions and to learn more about the ecology and evolution of the endosymbionts [33–36]. Here we reconstructed the complete genomes of four endosymbionts of the spider *O. gibbosus* including two different *Wolbachia *, one ‘*Ca*. Tisiphia’ and one *Cardinium* genome. Together with the previously published genome sequence of ‘*Candidatus* Rhabdochlamydia oedothoracis’*,* we analysed coexistence patterns of the endosymbionts across different *O. gibbosus* populations using short-read sequence data. We then compared the endosymbiont genomes to their closest known relatives and studied nutrient provisioning pathways, secretion systems and potential effectors of the endosymbionts to better understand host–microbe and microbe–microbe interactions in this system. The complete and closed symbiont genomes also enabled us to have a closer look at transposable elements. By reconstructing their phylogenies, we provide a glimpse into the evolutionary past and found evidence for an early coexistence of the endosymbionts in the same hosts.

## Methods

### Endosymbiont genome assembly

To reconstruct the endosymbiont genomes, we used long-read (PacBio; W744xW766) and short-read (Illumina; W791, W815) sequencing libraries from the host *O. gibbosus* described previously [37]. Detailed information on the sequencing libraries is provided in Table S1. As references for the long-read alignments, we used metagenome-assembled genomes (MAGs) from the endosymbionts that were reconstructed from the short-read libraries. In addition, we used the contigs from the host-assembly that were classified as endosymbiont contigs using a custom Kraken database as described previously [37].

For *Cardinium* sp. cOegibbosus-W744×776, *Wolbachia* sp. wOegibbosus-W744×776A and *Wolbachia* sp. wOegibbosus-W744×776B we mapped long reads to the respective MAGs and contigs using minimap2 (v2.17) [38]. Finally, all mapped reads were merged, and duplicates were removed. As the coverage was too high for *Wolbachia *, the mapped reads were subsampled to a coverage of 70×. The final set of reads was then assembled using Unicycler (v0.4.6) [39]. The quality of the assemblies was checked by checkM (v1.0.18) [40] and visually inspecting the assembly graph [41]. The *Wolbachia* genomes were closed manually, and the assembly of *Wolbachia* sp. wOegibbosus-W744×776B was manually curated to remove misassembled regions.

For *Candidatus* Tisiphia sp. Oegibbosus-W744×776 we mapped long reads to the respective MAG and contigs using minimap2 (v2.17) [38] and short reads using bbmap (v37.61) (sourceforge.net/projects/bbmap/). Both long and short reads were merged and deduplicated afterwards. The final set of long and short reads was then used for a hybrid assembly using Unicycler (v0.4.6) [39]. The quality of the assembly was checked by checkM (v1.0.18) [40] and visually inspecting the assembly graph [41].

*Rhabdochlamydia oedothoracis* MAG OV001 was assembled using the paired-end (2×150 bp) short-read library ‘OV001_G’ (SRX9657682, see details of sequencing libraries used in this study in Table S1). ‘OV001_G’ is the ID of the individual used for sequencing, with ‘OV001’ being unique to the sequencing library, and thus used for strain designation. We first carried out a *de novo* assembly using MEGAHIT (v1.1.2) [42]. Afterwards, the contigs were binned using MetaBAT 2 (v2.15) [43]. The required bam file for binning was created using bowtie2 (v2.3.5.1) [44]. Finally, the qualities of all bins were checked with checkM (v1.0.18) [40]. The bin belonging to *Rhabdochlamydia* was used for a reassembly. For that purpose, we mapped the short-read library from Overmere_OV001 to the *Rhabdochlamydia* bin and the reference genome *R. oedothoracis* W744xW776 (GenBank accession number: CP075587-CP075588) using bbmap (v37.61) (sourceforge.net/projects/bbmap/). Afterwards all mapped reads were merged, and duplicates were removed. This set of reads was then used for a second round of *de novo* assembly and binning as described before. The quality of the final MAG was again checked with (v1.0.18) [40].

Origins of replication were detected using originx [45] and Ori-Finder [46]. The orientation of genome sequences was adjusted to previously sequenced *Wolbachia* and *Rickettsia* genomes [47].

### Abundance estimations of the endosymbionts

To estimate the abundance of the different endosymbionts in *O. gibbosus* individuals we used a dataset consisting of 16 short-read (Illumina) sequencing libraries obtained from male *O. gibbosus* individuals from six different sampling locations (Damvallei, *n*=4; Sevendonck, *n*=2; Pollismolen, *n*=2; Honegem, *n*=2; Overmeren, *n*=2; Walenbos, *n*=4) described previously [37]. Detailed information about the sequencing libraries is provided in Table S1. Illumina reads were right-tail clipped using a minimum quality of 20, and all reads shorter than 75 after this treatment were discarded using FASTX-Toolkit (http://hannonlab.cshl.edu/fastx_toolkit/). Reads with undefined nucleotides or left without a pair were removed using PRINSEQ [48]. Afterwards, the reads were mapped to the genome of *O. gibbosus* (GCA_019343175.1) using bbmap (v37.61; ‘-minid=99’) (sourceforge.net/projects/bbmap/). The unmapped reads were then mapped to the endosymbiont genomes, and the mapped reads to the single-copy gene elongation-factor alpha (EF1alpha) from *O. gibbosus* [37] (JTE90_028130) using Bowtie2 (v2.3.5.1) [44]. The median coverage for each endosymbiont genome and the mean coverage for EF1alpha was calculated per sample using samtools (v1.12) [49]. For ‘*Ca*. Tisiphia’, *Cardinium* and *Rhabdochlamydia* the coverage across the whole genome was used, while for *Wolbachia* A and *Wolbachia* B only the coverage of the respective accessory genes (*n*=319 *Wolbachia* A, *n*=353 *Wolbachia* B) was used. We used the coverage of the accessory genes for *Wolbachia* as the two genomes share several genes which would bias the abundance estimations as short reads from *Wolbachia* A would also map to *Wolbachia* B and vice versa. To calculate the abundance of each endosymbiont per sample, we normalized the median coverage of the endosymbionts by the mean coverage of EF1alpha. To get the number of endosymbionts by host cell we divided the abundance by the number of host chromosomes (*n*=2).

### Phylogenetic analyses

For the phylogenetic reconstructions of *Wolbachia* and *Cardinium* we downloaded representative genomes belonging to different groups from NCBI (Table S8). In the case of *Wolbachia *, *Anaplasma phagocytophilum* strain HZ and *Ehrlichia canis* strain Jake were used as outgroups, and for *Cardinium *, *Amoebophilus asiaticus* strain 5a2 was used. rRNA proteins were extracted, and universally conserved ones selected. The protein sequences were individually aligned using MAFFT (v7.453; ‘--localpair’ ‘--maxiterate 1000’) [50]. Divergent and ambiguously aligned blocks were removed using Gblocks (v0.91b) [51]. The alignments were then concatenated using custom perl scripts and Bayesian inference was performed using MrBayes (v3.2.7) [52], using the JTT+I+G4 substitution model. We ran two independent analyses with four chains each for 300 000 generations and checked for convergence (convergence diagnostic≤0.01).

For *Rickettsiaceae* we also downloaded representative genomes from NCBI and used *Megaira* sp. strain MegNEIS296, *Megaira* sp. strain MegCarteria, *Occidentia massiliensis* strain Os18, *Orientia tsutsugamushi* strain Boryong and *Orientia chuto* strain Dubai as outgroups (Table S8). We extracted rRNA proteins and selected universally conserved ones. The protein sequences were individually aligned using muscle (v3.8.31) [53] and the alignments were concatenated using AMAS [54]. The phylogeny was calculated with IQ-TREE 2 (v2.1.2; ‘-bnni’ ‘-alrt 1000’ ‘-m TESTNEW’ ‘--madd LG4X’ ‘-bb 1000’) using the JTTDCMut+F+R3 substitution model [55]. In order to verify if the endosymbionts belong to an existing clade or represent a new one, we calculated the average amino acid (AAI) and average nucleotide identity (ANI) for the endosymbiont and selected representative genomes using the method and thresholds described previously [56]. We did not exclude transposase genes for the ANI and AAI calculations. While a large amount of transposase genes might influence the ANI value, the AAI is regarded to be unbiased as only homologous genes are used for the calculation.

### Genome annotation and genome content analysis

The genome of ‘*Candidatus* Tisiphia’ sp. Oegibbosus-W744×776, *Rhabdochlamydia oedothoracis* MAG OV001, *Wolbachia* sp. wOegibbosus-W744×776A and *Wolbachia* sp. wOegibbosus-W744×776B were annotated using prokka (v 1.14.6; ‘--compliant’ ‘--kingdom Bacteria’) [57]. The genome of *Cardinium* sp. cOegibbosus-W744×776 was annotated using Prokka (v1.14.6; --kingdom Bacteria) [57], Infernal (v1.1.4) with the bacterial subset of Rfam (v14.2; --cut_tc –mid) [58], and tRNAscan-SE (v2.0.9; -B -I –isospecific) [58]. The non-ribosomal peptide synthase (NRPS) gene was identified from the draft prokka annotation, and a more detailed annotation was done using AntiSMASH (v6.1) as implemented through the webserver [59].

For the genome content analysis of ‘*Ca*. Tisiphia’, *Cardinium* and *Wolbachia,* we mapped all proteins against the eggNOG database (v5.0) [60] using EggNOG-mapper (v2.1.0) [61] to assign them to existing gene families. For *Rhabdochlamydia,* we used the eggNOG database (v4.5.1) [62] and EggNOG-mapper (v1.0.1, ‘-d bact’) [63]. For all unmapped proteins we performed an all-against-all blastp [64] search and clustered proteins with an e-value <0.001 *de novo* with SiLiX (v1.2.11) [65] with default parameters. For functional annotation we used eggNOG (v5.0; v4.5.1) [60, 62], and blastp against the NCBI nr database for the *de novo* gene families. For analysis of the accessory genomes, we removed all proteins shorter than 100 amino acids in order to remove ghost coding sequences (CDSs) and remnants of transposases. To show the co-localization of transposase genes, accessory genes and regions covered by *R. oedothoracis* MAG OV001, we extracted the loci of transposase and accessory genes using a custom python script and identified homologous regions between *R. oedothoracis* W744×776 and MAG OV001 using mummer (v3.0; ‘nucmer --mum’; ‘delta-filter -qr’) [66]. Afterwards, the genome was plotted with circos [67]. To link breaks in the synteny between *R. oedothoracis* W744×776 and *R. oedothoracis* MAG OV001 to transposases, contigs showing rearrangements were selected using mauve. Homologies between the contigs and the reference genome were identified using blastn and the alignments and annotations were visualized using R (v4.0.3) [68] and the genoPlotR package (v0.8.11) [69].

Secretion systems were identified by BLASTP (‘-evalue 1e-3’) [64] against selected representatives [* Cardinium* sp. cEper1 (GCA_000304455.1), *Tisiphia* sp. RiCNE (GCA_002259525.1), *Wolbachia* sp. wMel (GCA_000008025.1)). The protein sequences were then individually aligned using MAFFT (v7.453; ‘--maxiterate 1000’) [50]. To investigate the presence of eukaryotic-like domains in the endosymbiont genomes, we screened their proteomes and those of selected representatives [* Amoebophilus asiaticus* 5a2 (GCA_000020565.1), *Rhabdochlamydia porcellionis* (GCA_015356815.2), *Rickettsia bellii* RML369-C (GCA_000012385.1), *Rickettsia rickettsii* Iowa (GCA_000017445.3)] using InterProScan (v5.53–87.0; ‘-appl Pfam’ ‘-dp’) [70]. The presence of vitamin and essential amino acid biosynthesis pathways was checked using GhostKOALA (v2.2) [71] and the ‘reconstruct’ function of KEGG Mapper [72]. Biotin genes in the *Wolbachia* genomes were identified as described for the secretion systems [* Wolbachia* sp. wNfla (GCA_001675695.1), *Wolbachia* sp. wNleu (GCA_001675715.1), *Wolbachia* sp. wLug (GCA_007115045.1), *Wolbachia* sp. wCle (GCA_000829315.1), *Wolbachia* sp. wstri (GCA_007115015.1)]. Toxin–antitoxin systems were identified by text search (grep -i ‘toxin\|antidote’) against the EggNOG-mapper and Prokka annotations. The results were checked by BLASTP against the NCBI nr database.

### Analysis of shared transposases

For the phylogenetic analysis of shared transposases we first clustered all genes annotated as transposases by prokka [57] into gene families using SiLiX (v1.2.11) [65]. For each gene family that was shared by two or more endosymbionts we searched for homologous sequences using the blastp function of ISfinder [73] and created a multiple sequence alignment with MAFFT (v7.453; ‘--maxiterate 1000’) [50]. Afterwards, the alignments were manually checked and sequences showing clear signs of degradation either on the 3′ or 5′ end were removed. We took care to only remove transposase sequences that seemed degraded (i.e. pseudogenized) in comparison to otherwise highly identical genes in order to keep the dataset clear of sequences that might be under different selective pressures. Finally, the alignments were trimmed using BMGE (v1.12) [74] and used for phylogenetic reconstruction using iqtree2 (v2.1.2; ‘-bnni’ ‘-alrt 1000’ ‘-m TESTNEW’ ‘-bb 1000’ ‘-mset LG’ ‘-madd LG+C10,LG+C20,LG+C30,LG+C40,LG+C50,LG+C60’ ‘-keep-ident’ ‘-wbtl’) [55]. For transposase sequences showing a clear sister-clade relationship in the *de novo* trees and belonging to the same eggNOG gene family, we reconstructed phylogenetic trees using gene families based on the eggNOG database (v5.0) [60] and EggNOG-mapper (v2.1.0) [61]. For this, we added the protein sequences from the respective gene families from the eggNOG database (v5.0) [60] to the endosymbiont gene families. For each gene family we then calculated multiple sequence alignments, curated them and reconstructed phylogenies as described above.

### Prediction of horizontal gene transfers

We used HGTector (v2.0; ‘--evalue 1e-20 --identity 30 --coverage 40’; and for *‘Ca. Tisiphia’* ‘--self-tax 114295 --close-tax 780’) [75] to predict horizontal gene transfer (HGT) events in the endosymbiont genomes. To keep the analysis conservative, we excluded all genes annotated as transposable elements (TEs) (IS elements, phages, introns) or genes that are part of TEs (integrases, reverse transcriptases) from the output. We also excluded genes predicted to be transferred from members of the phylum *Chlamydiae* for *Rhabdochlamydia oedothoracis* W744×776 and genes predicted to be transferred from members of the family *Rickettsiaceae* for *Candidatus* Tisiphia sp. Oegibbosus-W744×776 as we focused our analysis on HGTs between distantly related groups.

### SNP calling

For calling genetic variants we used the same short-read sequencing libraries as described for the abundance estimations, but we excluded all samples from the Walenbos population as the endosymbiont genomes were reconstructed from this population, and we excluded all samples with a coverage <30. Afterwards, we applied breseq (v0.36.1) [76] to call genetic variants present in the sequencing libraries in comparison to the reference genomes. We excluded all variants with a frequency <1 and variants affecting transposases or introns from the output and calculated the total number of mutations/kb and relative abundances of variant types per sample.

### Statistical analysis

All statistical tests and data analysis were performed in R (v4.0.3) [68] and visualized using ggplot2 (v3.3.3) [77]. Hierarchical clustering was done using the ‘hclust’ function (‘stats’ package v4.0.3) [68] and a distance matrix created from symbiont abundance data using the ‘dist’ function (‘stats’ package v4.0.3) [68].

## Results and discussion

### Co-occurrence of endosymbionts in different host populations

Co-infections of multiple reproductive symbionts of the groups *Wolbachia *, *Cardinium* and *Rickettsiaceae* within the same individual appear to happen only rarely. To get an idea about the distribution of the endosymbionts among *O. gibbosus* individuals from geographically distinct populations, we first reconstructed the endosymbiont genomes from sequence data obtained from the host *O. gibbosus* by Hendrickx *et al*. in 2021 (Table S2). We calculated the relative abundance of the symbionts and symbiont load (i.e. number of symbiont genomes per diploid host cell) by mapping short-read sequence data obtained from adult male *O. gibbosus* individuals to the reference genomes of the endosymbionts.

**Fig. 1.**
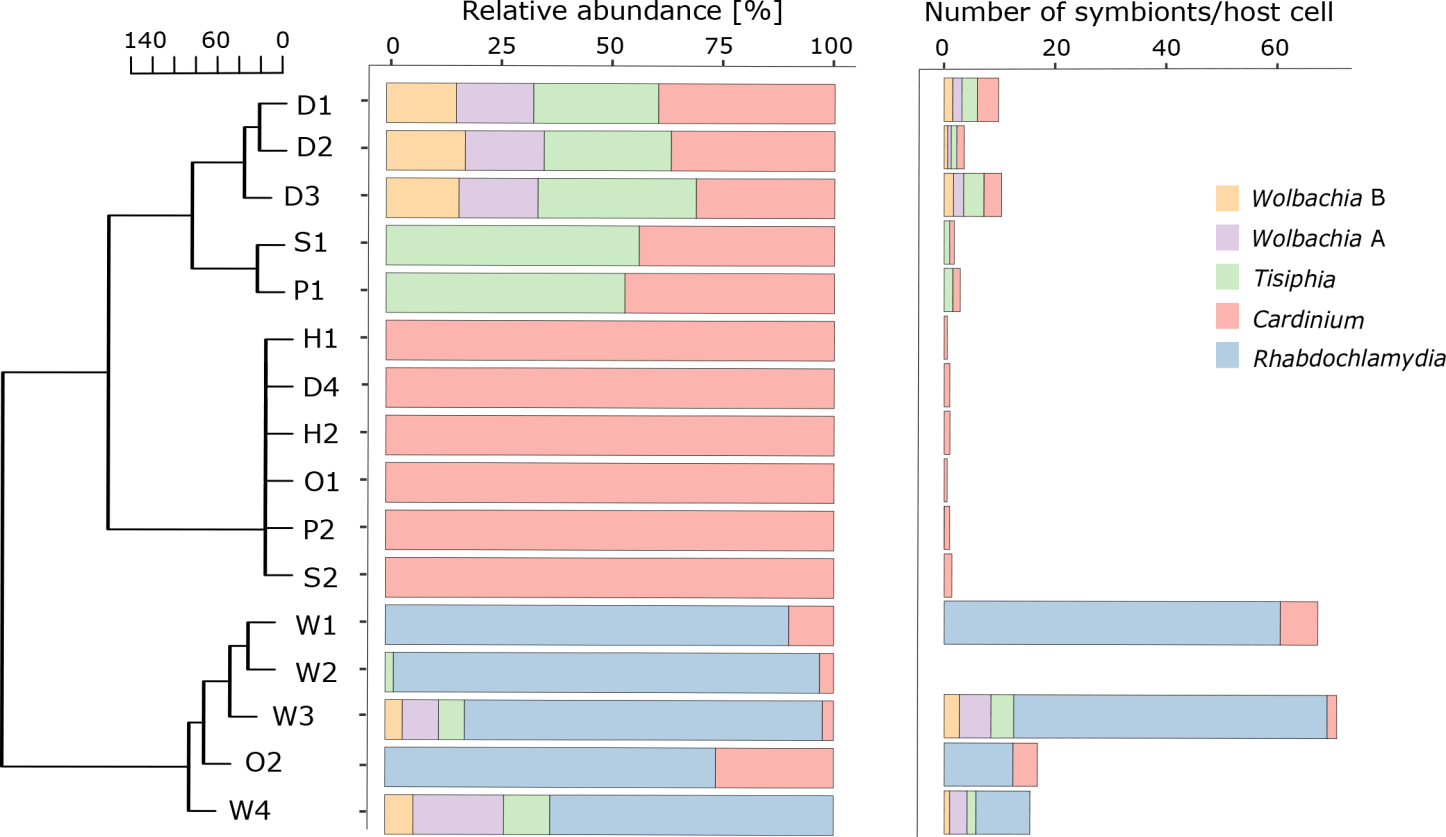
Relative abundance of the endosymbionts and symbiont load in male *O. gibbosus* individuals of different populations. Relative abundances and symbiont load (i.e. number of symbionts per diploid host cell) were calculated based on the coverage of the endosymbionts normalized by the coverage of elongation factor alpha of *O. gibbosus*. The samples were grouped using hierarchical clustering based on a Euclidean distance matrix. Samples were obtained from different locations across Belgium: D=Damvallei (*n*=4), S=Sevendonck (*n*=2), P=Pollismolen (*n*=2), H=Honegem (*n*=2), O=Overmeren (*n*=2), W=Walenbos (*n*=4).

We observed co-infections with multiple endosymbionts to occur frequently in *O. gibbosus* as 10 out of 16 spiders from six different locations across Belgium are infected with two or more endosymbionts. Further, we noted large variability regarding the distribution of the endosymbionts even between *O. gibbosus* individuals of the same population (Fig. 1). *Cardinium* is the most prevalent symbiont occurring in almost all sequenced individuals (94%), while *Rhabdochlamydia* is limited to the Walenbos and Overmeren populations (Fig. 1). Further, except for individuals containing *Rhabdochlamydia *, the symbiont load is generally low (3.10 symbionts per host cell on average) (Fig. 1). In spiders infected with *Rhabdochlamydia* the number of endosymbionts increases to 42.5 on average, with *Rhabdochlamydia* being the most abundant member of the community. Its high abundance could point towards a pathogenic rather than mutualistic or commensal role of *Rhabdochlamydia* in the spiders, as suggested for other members of the genus [24, 26, 27]. In addition, we found the two *Wolbachia* symbionts co-infecting individuals in two different populations. Interestingly, we found *Wolbachia* in two male spiders from the Walenbos population (Fig. 1) that was previously shown to be affected by male-killing *Wolbachia* [78]. As suggested earlier, this could hint towards an incomplete male-killing allowing some male offspring to survive [79]. It is of note that we only analysed male spiders, and thus the distribution of the endosymbionts might differ in females.

To get an idea about the genetic variability between the endosymbionts of different populations we analysed genomic variations, i.e. SNPs, insertions/deletions (indels) and substitutions, by mapping short-read sequence data from individual spiders against the reference genomes obtained from the Walenbos population. Whereas we find only low numbers of variants (0.1 mutations kb^–1^ on average) for *Cardinium* and *Rhabdochlamydia* suggesting nearly clonal symbiont populations, the ‘*Ca*. Tisiphia’ symbionts are considerably more heterogeneous (7.1 mutations kb^–1^ on average) (Fig. S1). However, as this type of analysis can presently be performed with data from three different host populations only, future in-depth analysis with more comprehensive datasets will reveal whether the observed differences in genetic variability between the symbionts are a more general feature.

## Phylogeny and genomic repertoire of the endosymbionts

### Rhabdochlamydia

The genome of the *Rhabdochlamydia* symbiont of *O. gibbosus* was previously described [80]. It revealed a high similarity to its closest relatives *R. porcellionis* and ‘*Ca*. R. helvetica’, infecting crustacean and arachnid hosts, respectively [23, 25]. Unlike other *Rhabdochlamydia *, the genome of ‘*Ca*. R. oedothoracis’ contains a large number of TEs making up about 23 % of the genome and leading to the hypothesis that its genome is currently undergoing genome size reduction [80]. Here, we compare the published genome of ‘*Ca*. R. oedothoracis W744×776’ (hereafter *R. oedothoracis*) isolated from *O. gibbosus* from the Walenbos population with a medium-quality MAG (*sensu* [81]) (Table S2) newly reconstructed from *O. gibbosus* from the Overmeren population (‘*Ca*. R. oedothoracis OV001’, hereafter MAG rhOegib-Ov). According to the AAI (99.47 %) and ANI (99.79 %) the two genomes belong to the same species [56].

**Fig. 2.**
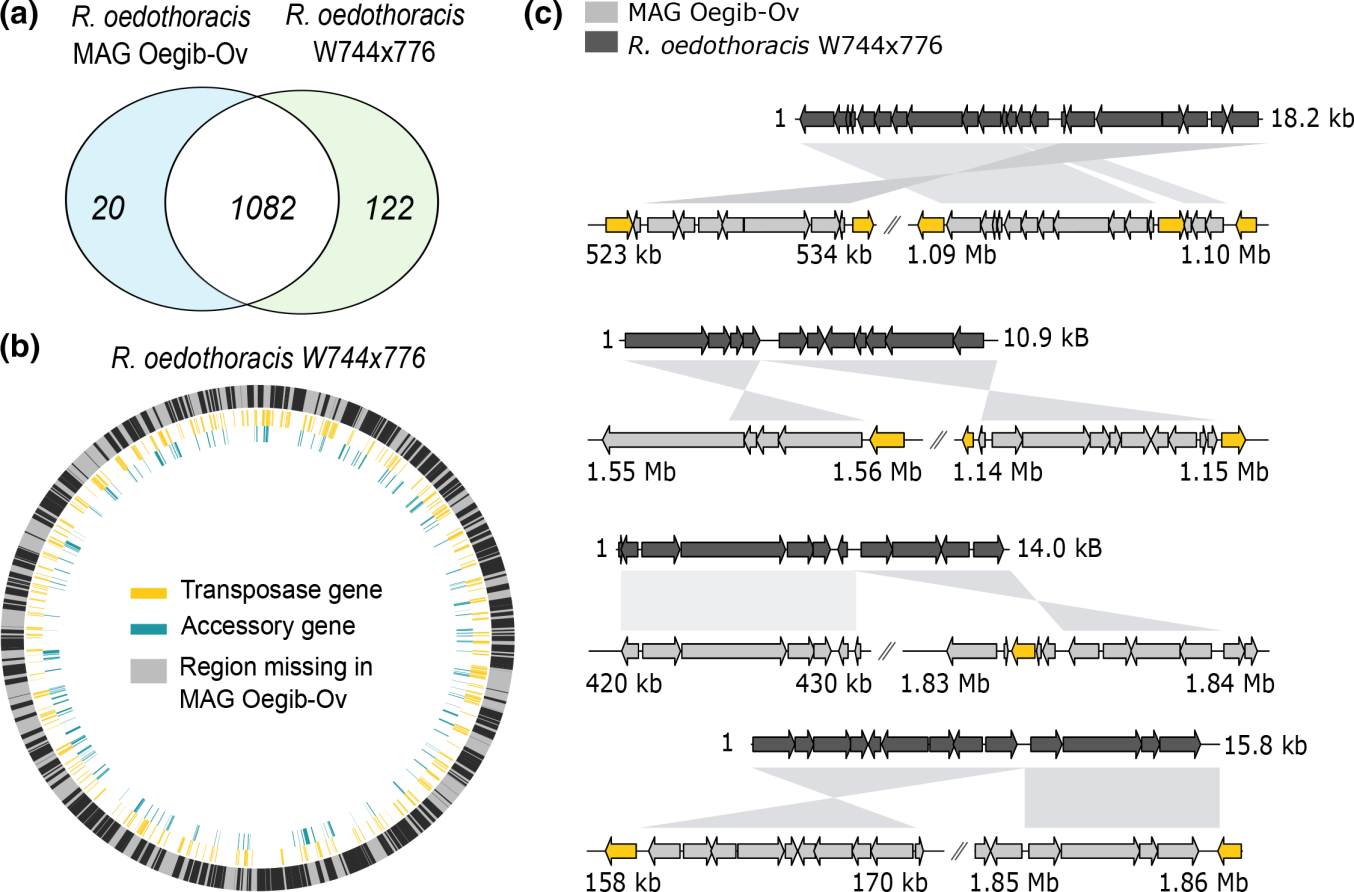
Genome comparison of two *Rhabdochlamydia oedothoracis* strains*,* W744×776 and MAG rhOegib-Ov. (**a**) Genome content comparison based on eggNOG and *de novo* clustered gene families (OGs). For the analysis only proteins with a minimum length of 100 aa were used. (**b**) Genome alignment of *R. oedothoracis* W744×776 and MAG rhOegib-Ov. Regions present in MAG rhOegib-Ov are shown in black, annotated transposases in the genome of *R. oedothoracis* W744×776 are shown in yellow, and accessory genes of *R. oedothoracis* W744×776 are shown in cyan. (**c**) Selected regions representing genomic rearrangements and co-localized transposase genes between *R. oedothoracis* W744×776 and MAG Oegib-Ov. Transposase genes are shown in yellow and other CDSs in shades of grey.

To compare the two genomes we first clustered all predicted proteins into orthologous groups (OGs; corresponding to gene families) using either eggNOG (v4.5) [62] or SiLiX (v1.2.11) [65], where SiLiX was used for *de novo* clustering of genes not annotated in eggNOG (v4.5) [62]. For the best studied members of the phylum *Chlamydiae *, namely members of the family *Chlamydiaceae *, it has previously been shown that strains of the same species are highly similar in gene content and genome synteny [82–84]. We observed a similar pattern for *R. oedothoracis*, as the two genomes share most of their gene families (Fig. 2a). The number of accessory gene families that are specific for one of the genomes is significantly larger for *R. oedothoracis*. However, we observed that most of the genes that are only present in the genome of *R. oedothoracis* but missing in MAG rhOegib-Ov are located in close proximity to transposase genes (Fig. 2b). This suggests that those genes were probably not assembled in MAG rhOegib-Ov due to the presence of multiple copies of diverse transposases. Such assembly challenges in metagenomic data highlight the importance of available complete genome sequences especially for groups known to include many TEs. Our comparative analysis further confirmed the involvement of TEs in genome rearrangements and genome evolution in *Rhabdochlamydia oedothoracis* as suggested earlier [80], as the location of transposase genes tends to correspond to breakages in synteny between the two genomes (Fig. 2c).

### Cardinium

**Fig. 3.**
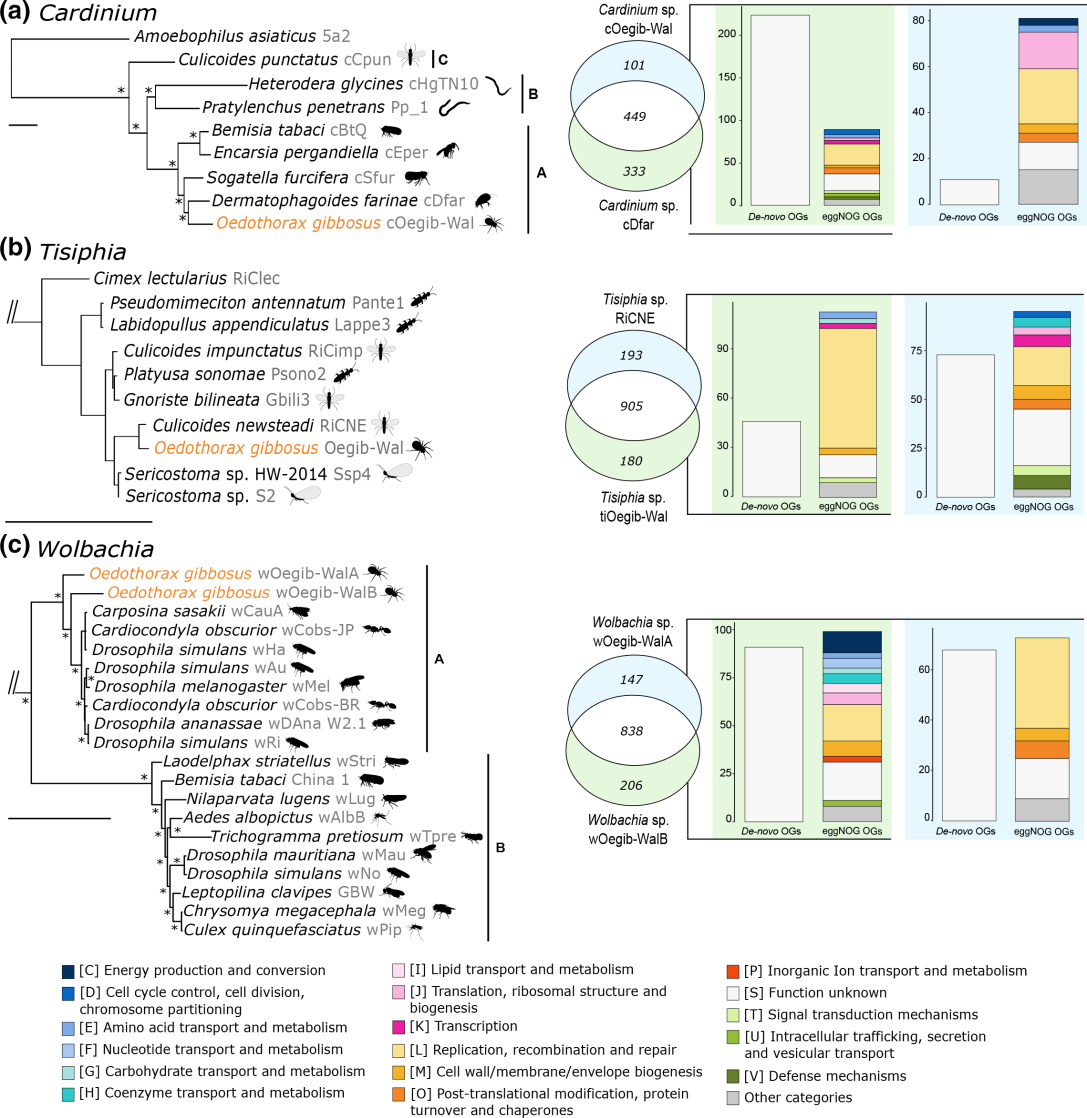
Relationship of the *Cardinium *, ‘*Candidatus* Tisiphia’ and *Wolbachia* endosymbionts of *O. gibbosus*, and genome comparison with their closest relatives. (**a**) Ribosomal protein gene phylogeny of the genus *Cardinium *. Bar, 0.07 substitutions per position in the alignment. Asterisks mark branches with a posterior probability of 1. The tree was rooted using the amoeba symbiont *Amoebophilus asi* aticus 5a2 as an outgroup. (**b**) Ribosomal protein gene phylogeny of the genus ‘*Candidatus* Tisiphia’. Bar, 0.1 substitutions per position in the alignment. The tree was rooted using *Orientia* sp. and ‘*Candidatus* Megaira’ sp. as an outgroup. All branches have a support value ≥95. (**c**) Ribosomal protein gene phylogeny of the genus *Wolbachia *. Bar, 0.07 substitutions per position in the alignment. Asterisks mark branches with a posterior probability of 1. The tree was rooted using *Anaplasma phagocytophilum* and *Ehrlichia canis* as an outgroup. Host names are indicated at the leaves. For clear presentation only selected clades are shown in the trees, the complete dataset used to reconstruct the trees is shown in Table S8 and the complete trees are provided in the zenodo repository. Genome comparison as Venn diagrams and composition of accessory genomes based on eggNOG and *de novo* clustered gene families (OGs) are shown next to the phylogenetic trees. Only unambiguously annotated COG categories were used, and only categories *n*>2 are shown; the rest are clustered into ‘Other categories’. For the analysis only proteins with a minimum length of 100 aa were used.

The genome of the endosymbiont *Cardinium* sp. cOegibbosus-W744×776 (hereafter cOegib-Wal) (Table S2) is 1.1 Mb in size and contains a high number of TEs, making up around 30 % of the genome. The ribosomal protein phylogeny places *Cardinium* sp. cOegib-Wal in the group A *Cardinium* (Fig. 3a) [85, 86], which encompasses *Cardinium* strains associated with insects and arachnids [85]. *Cardinium* sp. cOegib-Wal is nested in a clade with *Cardinium* symbionts of the rice bug *Stenocoris furcifera* (cSfur) and the mite *Dermatophagoides farinae* (cDfar) that seems to consist of three distinct species based on both ANI and AAI (Table S3). The sister clade comprises *Cardinium* symbionts of the whitefly *Bemisia tabaci* (cBtQ) and the parasitic wasp *Encarsia pergandiella* (cEper). Their genomes are very similar on the nucleotide level and based on their genome features even suggested to be strains of the same species [87]. However, this finding is not surprising as there is an ecological link between *Cardinium* sp. cBtQ and *Cardinium* sp. cEper: *E. pergandiella* is a common parasitoid of *B. tabaci*, while the hosts of the other *Cardinium* group A members are not ecologically related. The closest sequenced relative to *Cardinium* sp. cOegib-Wal is the widely distributed mite-associated *Cardinium* sp. cDfar [88] (Fig. 3a).

Our genome content analysis of *Cardinium* sp. cOegib-Wal and its closest relative *Cardinium* sp. cDfar revealed a reduced genomic repertoire of cOegib-Wal and a substantial fraction of gene families that is specific for the mite symbiont cDfar (Fig. 3a). The putative function of these gene families was inferred from eggNOG (v5.0) [60] and BLASTP [64] against the NCBI-nr database. Most represent hypothetical proteins (COG category S and *de novo* OGs) with homologes in other *Cardinium* strains, suggesting a loss of those genes in *Cardinium* sp. cOegib-Wal. The presence of COG categories involved in metabolic processes (C, D, E, J, K, O) in the accessory genomes of cOegib-Wal and cDfar probably indicates differences in the basic metabolism while the presence of COG categories involved in defence mechanisms, secretion, signal transduction and cell wall/membrane biosynthesis (V, U, T, and M) probably indicates adaptation to different hosts and environments (Fig. 3a).

Notably, the genome of cOegib-Wal encodes a large NRPS cluster consisting of a large modular protein and its helper proteins. NRPSs are widespread in prokaryotes and eukaryotes and of particular interest due to their antimicrobial and antifungal properties [89]. The NRPS found in cOegib-Wal is predicted to synthesize a short ‘TNTXXLP’ peptide, for which its action remains unknown (Fig. S2). From currently fully sequenced *Cardinium* genomes, only the nematode-infecting strain cHgTN10 contains an NRPS gene cluster. However, these two NRPSs are not homologous.

### *Candidatus* Tisiphia

The closed genome of ‘*Candidatus* Tisiphia’ sp. Oegib-W744×776 (hereafter *Tisiphia sp*. tiOegib-Wal) is 2.6 Mb in size (Table S2) and consists of 28 % TEs. According to the ribosomal protein phylogeny the genome belongs to the recently proposed genus ‘*Ca*. Tisiphia’ (formerly Torix group *Rickettsia *), a sister group of the genus *Rickettsia* [15] (Fig. 3b). The phylogenetic placement is further corroborated by a 16S rRNA phylogeny (Supplementary methods, Fig. S3). Its closest sequenced relative is ‘*Ca*. Tisiphia’ sp. RiCNE (formerly *Rickettsia* sp. RiCNE, hereafter *Tisiphia sp*. RiCNE), a symbiont of the midge *Culicoides newsteadi* (Fig. 3b) [90]. According to the AAI (94.11 %) and ANI (97.00 %) scores, the two genomes belong to the same species. We could not observe a pattern regarding phylogeny and host range in ‘*Ca*. Tisiphia’ (Figs 3b and S3). Further, *Tisiphia* sp. RiCNE and *Tisiphia* sp. tiOegib-Wal are strains of the same molecular species but were isolated from phylogenetically distinct hosts. Together, this could indicate a propensity for horizontal transmission of this ‘*Ca*. Tisiphia’ species between host species.

Although the two strains share most of their gene families, about 17 % of the gene families are unique to either one of them (Fig. 3b). Most of the gene families missing in *Tisiphia* sp. tiOegib-Wal are hypothetical proteins with close homologes in other *Rickettsiaceae,* suggesting a loss of those genes in *Tisiphia* sp. tiOegib-Wal. Furthermore, *Tisiphia* sp. tiOegib-Wal seems to have a more reduced genomic repertoire, with its accessory gene families falling into only a few different functional categories (Fig. 3b) and most of them being transposases and genes for their maintenance (COG category L). Interestingly, only a few accessory gene families are linked to host interaction (Categories T, M) (Fig. 3b) although the two strains were isolated from very different hosts.

### Wolbachia

We were able to reconstruct two different closed *Wolbachia* genomes (hereafter *Wolbachia* sp. wOeGib-WalA and *Wolbachia* sp. wOeGib-WalB) from *O. gibbosus*. According to the ribosomal protein gene phylogeny (Fig. 3c, Table S8), AAI and ANI (89 % and 93 %, respectively; Figs S4 and S5, Table S5), the two genomes represent separate lineages falling at the base of supergroup A of *Wolbachia *. Compared to the AAI/ANI of other supergroup A and B *Wolbachia* they still belong to supergroup A and do not form new supergroups (Figs S4 and S5, Table S5). Both genomes show a substantial fraction of TEs (13 % wOeGib-WalA, 12 % wOeGib-WalB) consistent with reports about other *Wolbachia* genomes [91, 92].

AS previously shown for other *Wolbachia* lineages, *Wolbachia* sp. wOeGib-WalA and *Wolbachia* sp. wOeGib-WalB share most of their gene content (Fig. 3c) [93]. Most accessory gene families in both genomes are hypothetical proteins (Fig. 3c; *de novo* OGs, COG category S) with close homologes in other *Wolbachia* suggesting a differential loss in one of the genomes. *Wolbachia* sp. wOeGib-WalB seems to have a larger and more diverse genetic repertoire as there are different functional categories represented in its accessory genome (Fig. 3c). In *Wolbachia* sp. wOeGib-WalA, on the other hand, most gene families with an assigned function fall into COG category L, which includes transposases and genes for their maintenance.

## Interactions of the endosymbionts with the host

### Reproductive manipulation

Although members of all three groups, *Cardinium *, *Wolbachia* and *Rickettsiaceae *, were shown to be able to manipulate their hosts' reproduction [3–7], a previous study only found a correlation between the presence of *Wolbachia* and female-biased offspring in *O. gibbosus* [78]. As the number of male and female eggs was similar in sex-biased and unbiased breeds, male-killing was suggested to cause the sex ratio bias [78]. *Wolbachia *-induce*d* sex ratio bias is thought to be mainly caused by the products of the *wmk* and the *cifA* and *cifB* genes [94]. Homologues of the *wmk* gene are present in all known male-killing *Wolbachia* strains [94], and there is growing evidence that this gene has a crucial role in male-killing [95]. We identified *wmk*-like genes in both *Wolbachia* genomes from *O. gibbosus* (WOOEGIBXXXB_1017, WOOEGIBWALA_1209). These genes are most similar to the homologues in the *Wolbachia* sp. wInn symbiont of *Drosophila innubila* (wOegib-WalA) and *Wolbachia* sp. wBif infecting the moth *Drepanogynis bifasciata* (wOegib-WalB). However, recent evidence has shown that transgenic expression of the wInn or wBif *wmk* homologues in *Drosophila melanogaster* does not recapitulate the male-killing phenotype [95]. Nonetheless, given that the genes were expressed without the presence of the corresponding *Wolbachia* and in a non-native insect host, the male-killing phenotype of these *wmk* homologues cannot be ruled out in their native hosts. The *cifA* and *cifB* genes known to be involved in cytoplasmic incompatibility are absent in *Wolbachia* sp. wOegib-WalA, but we were able to identify three clusters of highly similar, and probably paralogous, *cifA* and *cifB*-like genes in *Wolbachia* sp. wOegib-WalB (Fig. S6). However, these genes are quite divergent to *cifA* and *cifB* genes of other *Wolbachia* strains (average sequence identity *cifA* 19 % and *cifB* 17%). Therefore, their actual role as cytoplasmic incompatibility factors remains to be tested.

## Amino acid and vitamin synthesis

All five endosymbionts of *O. gibbosus* have reduced metabolic capabilities (Table S4). None of them is capable of synthesizing essential amino acids, and except for *Wolbachia* none of them is able to produce B-vitamins (thiamine, riboflavin, pyridoxal-5-phosphate, coenzyme A, folic acids, biotin). This observation is consistent with *O. gibbosus* being a generalist predator of small insects and thus not being limited in nutrients by its diet. Interestingly, *Wolbachia* sp. wOegib-WalA and wOegib-WalB code for a complete biotin synthesis pathway (Table S4) which includes six genes (*bioC*, *bioH*, *bioF*, *bioA*, *bioD* and *bioB*) that are organized in a gene cluster. The genes show high sequence similarity to a complete biotin operon that was found in only five other *Wolbachia* strains and suggested to have been recently horizontally acquired by *Wolbachia* [96][97–100]. While the biotin operon is used by some *Wolbachia* strains to supplement their hosts’ vitamin B-deficient diet [101, 102], the diverse diet of *O. gibbosus* is generally not regarded to be limited in biotin. However, as there is no information about the biotin needs of the spider, the role of biotin production by *Wolbachia* for the system remains to be investigated.

## Toxin–antitoxin systems

Toxin–antitoxin (TA) systems are two-component systems composed of a stable toxin (protein) and its labile antitoxin (RNA or protein) [103], where most toxins interfere with protein biosynthesis [104]. Originally, TA systems were discovered as plasmid maintenance tools, killing daughter cells that lack a functional copy of the plasmid [105]. However, TA systems were subsequently identified on almost all bacterial chromosomes [105]. Although the function of these chromosomal copies is still unclear, it is hypothesized that they might regulate bacterial growth or operate in host cell manipulation [106].

The genome of *Tisiphia* sp. tiOegib-Wal contains three type-II TA modules: two DinJ/YafQ and one HigB/A. In type-II TA systems both the toxin and the antitoxin are small proteins that form a protein–protein complex resulting in the neutralization of the toxin. Further, there is one TA module with only low sequence similarity to any described TA system in other *Rickettsiaceae,* and three type-II toxins (YoeB, RatA, BrnT) and two antitoxins not arranged in modules (Table S6). Generally, it is unusual to find toxins without the respective antitoxin as it was previously shown that the overexpression of type-II toxins results in cell death in the absence of its antitoxin [106]. However, there are other described genomes only encoding toxins, suggesting the presence of unknown mechanisms underlying antitoxin activity and a more complex relationship between toxin and antitoxin activities at physiological expression levels [106]. For *Tisiphia* sp. tiOegib-Wal we could identify potential antitoxins for the YoeB toxins encoded at the 5′ ends of the toxins. We found a similar module also in the genome of *Rickettsia felis,* and the putative antitoxin has close homologues in other *Rickettsia* and *Orientia *. However, there is no homology to the described antitoxin of YoeB, YefM.

The genome of *Wolbachia* sp. wOegib-WalB contains three copies of toxin RelE (Table S6) which is part of the RelEB type-II TA system also found in other *Wolbachia* genomes [107]. The three RelE copies are not orthologous according to eggNOG but contain a RelE domain. Nonetheless, we found no evidence for the presence of the corresponding antitoxin RelB that is present in other *Wolbachia* genomes.

### Secretion systems and potential effectors

Secretion systems are used by bacteria to export substrates out of the bacterial cell. These so-called effectors serve to communicate with the environment and in the case of intracellular bacteria with their host cells [101]. For the endosymbionts of *O. gibbosus* we could identify different secretion systems that are well described for the respective clade. *R. oedothoracis* encodes, like all known members of the phylum *Chlamydiae *, a complete type III secretion system [22, 80, 108]. The genome of *Cardinium* sp. cOegib-Wal comprises homologes of all 15 genes encoding the phage-derived type VI-like secretion system described previously in the amoeba symbiont *Amoebophilus asiaticus* [102, 109]. *Tisiphia* sp. tiOegib-Wal, *Wolbachia* sp. wOegib-WalA, and *Wolbachia* sp. wOegib-WalB encode all components of the conserved *Rickettsiales* type IV secretion system [90, 110, 111]. In addition, the genome of *Tisiphia* sp. tiOegib-Wal contains the *tra* gene clusters that encode a conjugative DNA‐transfer element called RAGE (Rickettsiales Amplified Genetic Element) and previously described in several *Rickettsia* genomes [90, 112–115].

Effectors of intracellular bacteria often harbour domains that have a high sequence similarity with eukaryotic proteins [116]. Proteins including such eukaryotic-like domains target and manipulate different key cellular processes and are used to interact with the host immune response [116, 117]. Further, a correlation between the bacterial lifestyle and the number of eukaryotic-like domains was suggested, where symbionts of microbial eukaryotes, such as amoeba, tend to encode a higher number of proteins with eukaryotic-like domains than free-living bacteria and intracellular bacteria of multicellular hosts [116]. However, the correlation was only systematically analysed for *Legionella* and *Chlamydiae *. To investigate potential effectors in the symbionts of *O. gibbosus* we screened their proteomes for known eukaryotic domains.

**Fig. 4.**
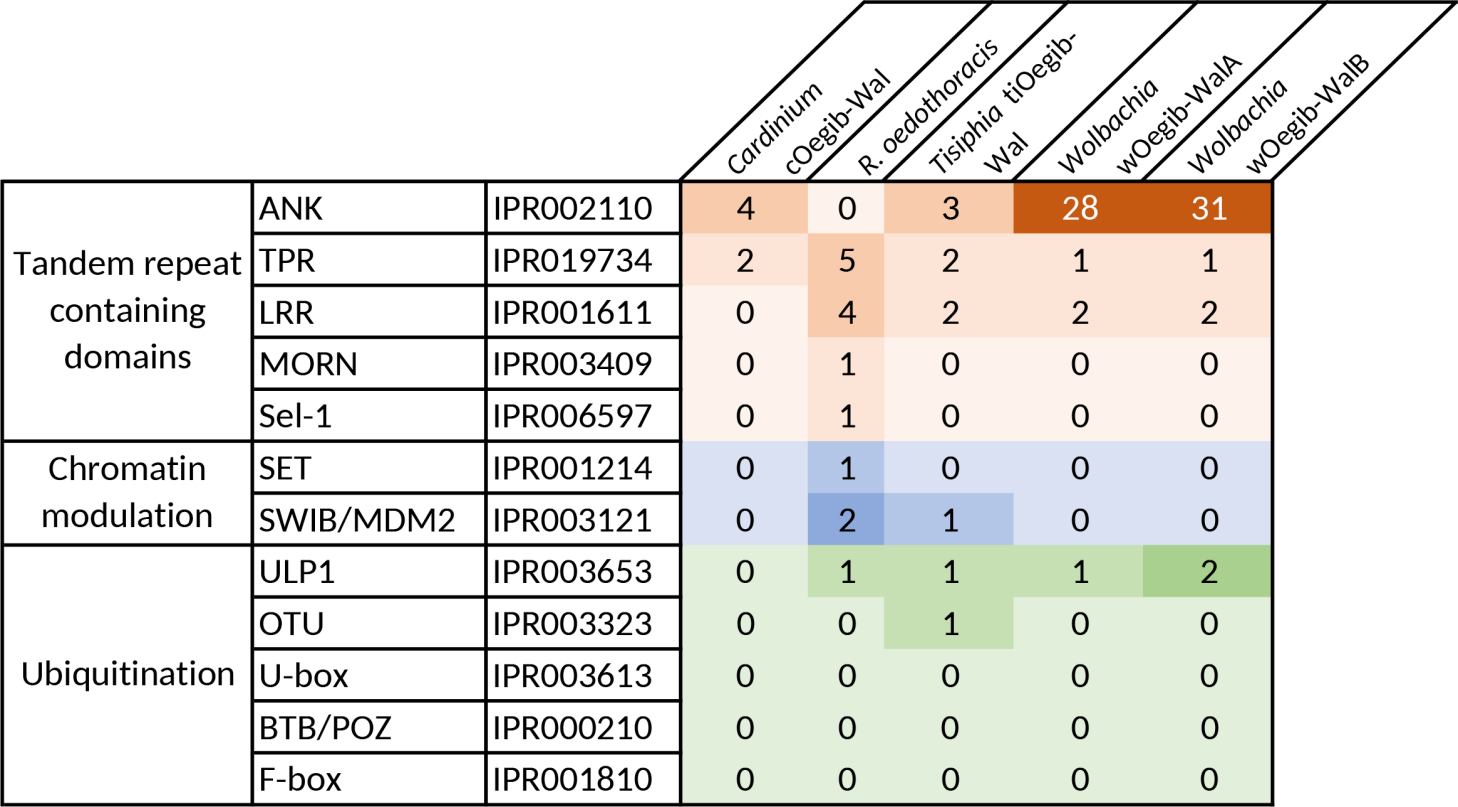
Eukaryotic-like domain-containing proteins of the endosymbionts of *O. gibbosus*. The domains were identified with InterProScan (v5.53–87.0) [70]. The colours indicate the different categories of eukaryotic-like domains and the intensity of the colour indicates the number of domains present in the respective genome.

The genome of *Cardinium* sp. cOegib-Wal contains only few ankyrin (ANK)- and tetratricopeptide repeat (TPR)-containing proteins (Fig. 4). ANK and TPR repeats are part of the so-called tandem-repeat domains that mediate protein–protein and protein–nucleic acid interactions in eukaryotes [116]. The presence of ANK- and TPR-containing proteins were also described for other group A *Cardinium* strains [87, 118]. *R. oedothoracis* encodes proteins with tandem-repeat domains, proteins with chromatin modulation domains, and a protein with a ubiquitination domain (Fig. 4). Proteins including chromatin modulation domains are able to influence host gene expression, and ubiquitin is a post-translational modification that mediates various protein interactions in eukaryotes. The genome of *Tisiphia* sp*. tiOegib-Wal* also contains proteins with all three types of domains (Fig. 4). The two *Wolbachia* genomes wOegib-WalA and wOegib-WalB contain proteins with domains involved in ubiquitination and a high number of ANK repeat-containing proteins (Fig. 4). This is in line with the number of ANK repeat-containing proteins found in other arthropod-infecting *Wolbachia* strains [119, 120]. So far no correlation could be found between the number of ANK repeat-containing proteins and the phenotype of *Wolbachia,* i.e. cytoplasmic incompatibility or male-killing [119]. It is of note that apart from proteins including eukaryotic-like domains, there is a variety of other effectors that are not discussed here.

### Transposases indicate horizontal transmission and past coexistence of the endosymbionts

Our analysis revealed that the genomes of all five endosymbionts of *O. gibbosus* contain a high number of TEs, especially insertion sequences (ISs). This simplest form of TEs consists only of a transposase gene flanked by inverted repeats [121]. ISs are mobile elements that can spread within a genome independent of cellular replication and are known to be major drivers of bacterial genome evolution and diversification [122]. They are involved in genome size reduction by inactivation of genes under relaxed selection [123], as suggested for *Rhabdochlamydia* [80]. ISs can also move between genomes of different species by means of HGT and thereby mediate the exchange of genetic material across species [122]. The presence of large numbers of mobile elements is common in genomes of facultative intracellular and extracellular bacteria but was regarded to be limited or absent in ancient obligate intracellular bacteria that thrive in a restricted niche with limited interaction with other bacteria [124, 125]. However, according to the ‘intracellular arena hypothesis’, obligate intracellular bacteria that switch hosts do indeed come into contact with other bacteria and thus can accumulate higher numbers of mobile elements in their genomes than those that are strictly bound to one host [125]. This hypothesis could also explain the high number of TEs found in the spider endosymbionts as even though the genera *Cardinium *, *Wolbachia* and *Rickettsiaceae* consist of obligate and mainly maternally transmitted endosymbionts, horizontal transmissions occur in all of them [14, 126–133]. This is also documented by the diverse co-occurrence patterns within and between populations observed here and elsewhere (Fig. 1) [21].

**Fig. 5.**
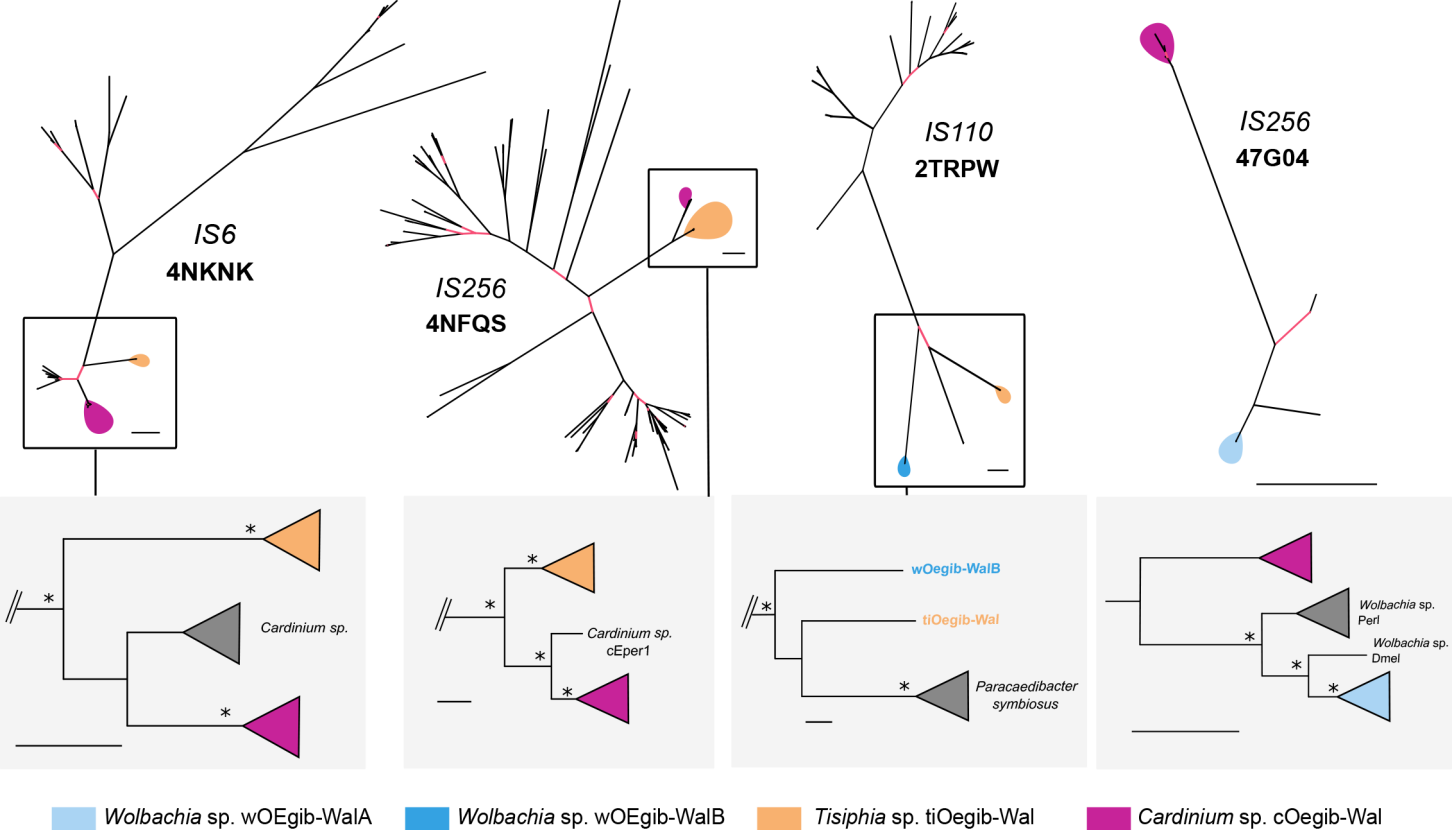
Unrooted phylogenies of shared transposases of the endosymbionts. Branches with bootstrap values >95 are indicated in black, while branches with lower bootstrap values are shown in red. The trees are labelled with the respective IS-family and eggNOG OG. Below each phylogeny, mid-point-rooted trees of selected clades are shown. Branches with support values >95 are indicated with asterisks. Further details on the phylogenies can be found in Table S7. Note that for the trees only complete sequences were used, and thus in some cases not all endosymbionts stated in Table S7 are represented in the trees. Bar, 0.1 substitutions per position in the alignment.

As TEs are frequently exchanged between bacteria sharing an environmental niche [134, 135], we used TE gene trees to infer whether the spider endosymbionts recently shared TEs or have coexisted and shared TEs in the past. For thisTo this end, we clustered all transposase genes into gene families using eggNOG (v5.0) [60]. For the families where two of more of the endosymbionts described here had TEs assigned (Table S7), we extracted all the sequences in the family and calculated phylogenies for each of them (Figs. 5 and S7). We found shared TEs between *Cardinium* sp. cOegib-Wal and *Tisiphia* sp*. tiOegib-Wal* (IS*6*, IS*256*), *Tisiphia* sp*. tiOegib-Wal* and *Wolbachia* wOegib-WalB (IS*110*), and a TE shared between ‘Ca. *Tisiphia*’, *Cardinium* and *Wolbachia* (IS*256*; Fig. 5, Table S7). A sister-clade relationship between the TEs in all phylogenetic trees and the presence of some TEs in other *Cardinium* and *Wolbachia* strains provide evidence for ancient evolutionary links and a possible shared niche in the past. Alternatively, an as yet-unknown or extinct common donor strain could be the source of the TEs. However, this would require two separate events of HGT, and given that some of these strains are common insect endosymbionts and sometimes found coexisting in individuals/populaitons, we find the transfer among endosymbiont strains a more likely scenario. Yet, we did not find any evidence for more recent transfers of transposase genes (Fig. 5). This finding is consistent with our results from screening the entire endosymbiont genomes for genes putatively acquired through HGT. While several HGT-derived candidates were predicted by HGTector [75] for all symbionts, we could not find support for any recent HGT events among the spider symbionts. Interestingly, however, TEs of *Wolbachia* wOegib-WalA and *Tisiphia* sp*. tiOegib-Wal* are related to a TE from the amoeba symbiont *Paracaedibacter symbiosus* (IS*110*; Fig. 5). This suggests an ancient coexistence of these amoeba and arthropod symbionts and is reminiscent of a similar case, in which a TE (ISRpe1) was found to be shared by *Wolbachia *, *Cardinium *, ‘Ca. *Tisiphia*’ and the amoeba symbiont *Amoebophilus asiaticus* [136]. Taken together, our phylogenetic analysis of transposase genes provides evidence for a shared niche of the *O. gibbosus* symbionts with amoeba symbionts and a co-occurrence during their evolutionary history. This analysis also shows that, due to their frequent transmission across species borders, TEs may provide a promising tool to reconstruct historical associations of microbes.

## Conclusions

In the current study we show that *O. gibbosus* is co-infected with up to five different endosymbionts that are heterogeneously distributed among different host populations. In contrast to other studies on arthropod endosymbionts that suggested co-infections with more than two endosymbionts occurred only rarely, we found that about 30 % of the 16 *O. gibbosus* individuals from six populations analysed here were infected with three or more endosymbionts. We were able to reconstruct four complete endosymbiont genomes present in the same host population. This offered us the unique opportunity to holistically explore this host–endosymbiont system on a genomic level. We identified all endosymbionts as facultative symbionts that are not essential for the survival of the spider and found indications for horizontal transmission of the endosymbionts, a route that is proposed for facultative symbionts as an alternative to the primarily vertical transmission [14, 126–133]. We detected large numbers of TEs in all endosymbiont genomes and showed that ancestral *Cardinium *, ‘*Ca*. Tisiphia’ and *Wolbachia* endosymbionts may have co-infected the same hosts in the past by using phylogenetic reconstructions of shared TEs. In summary, our findings contribute to broadening our knowledge about important groups of endosymbionts and set the stage for more in-depth analysis of host–endosymbiont interactions and interactions between different endosymbionts using the spider *O. gibbosus* as a model system.

## Supplementary Data

Supplementary material 1

Supplementary material 2

## References

[1] Giribet G, Edgecombe GD (2019). The Phylogeny and Evolutionary History of Arthropods. Curr Biol.

[2] Weinert LA, Araujo-Jnr EV, Ahmed MZ, Welch JJ (2015). The incidence of bacterial endosymbionts in terrestrial arthropods. Proc Biol Sci.

[3] Lawson ET, Mousseau TA, Klaper R, Hunter MD, Werren JH (2001). Rickettsia associated with male-killing in a buprestid beetle. Heredity (Edinb).

[4] Giorgini M, Monti MM, Caprio E, Stouthamer R, Hunter MS (2009). Feminization and the collapse of haplodiploidy in an asexual parasitoid wasp harboring the bacterial symbiont Cardinium. Heredity (Edinb).

[5] Giorgini M, Bernardo U, Monti MM, Nappo AG, Gebiola M (2010). Rickettsia symbionts cause parthenogenetic reproduction in the parasitoid wasp Pnigalio soemius (Hymenoptera: Eulophidae). Appl Environ Microbiol.

[6] Kaur R, Shropshire JD, Cross KL, Leigh B, Mansueto AJ (2021). Living in the endosymbiotic world of Wolbachia: A centennial review. Cell Host Microbe.

[7] Doremus MR, Stouthamer CM, Kelly SE, Schmitz-Esser S, Hunter MS (2022). Quality over quantity: unraveling the contributions to cytoplasmic incompatibility caused by two coinfecting Cardinium symbionts. Heredity (Edinb).

[8] Konecka E, Olszanowski Z (2019). A new Cardinium group of bacteria found in Achipteria coleoptrata (Acari: Oribatida). Mol Phylogenet Evol.

[9] Zchori-Fein E, Perlman SJ, Kelly SE, Katzir N, Hunter MS (2004). Characterization of a “Bacteroidetes” symbiont in Encarsia wasps (Hymenoptera: Aphelinidae): proposal of “Candidatus Cardinium hertigii.”. Int J Syst Evol Microbiol.

[10] Perlman SJ, Kelly SE, Hunter MS (2008). Population biology of cytoplasmic incompatibility: maintenance and spread of Cardinium symbionts in a parasitic wasp. Genetics.

[11] Li C, He M, Yun Y, Peng Y (2020). Co-infection with Wolbachia and Cardinium may promote the synthesis of fat and free amino acids in a small spider, Hylyphantes graminicola. J Invertebr Pathol.

[12] Li T-P, Zha S-S, Zhou C-Y, Gong J-T, Zhu Y-X (2020). Newly introduced Cardinium endosymbiont reduces microbial diversity in the rice brown planthopper Nilaparvata lugens. FEMS Microbiol Ecol.

[13] Hubert J, Nesvorna M, Pekar S, Green SJ, Klimov PB (2021). Cardinium inhibits Wolbachia in its mite host, Tyrophagus putrescentiae, and affects host fitness. FEMS Microbiol Ecol.

[14] Pilgrim J, Thongprem P, Davison HR, Siozios S, Baylis M (2021). Torix Rickettsia are widespread in arthropods and reflect a neglected symbiosis. Gigascience.

[15] Davison HR, Pilgrim J, Wybouw N, Parker J, Pirro S (2022). Genomic diversity across the Rickettsia and “Candidatus Megaira” genera and proposal of genus status for the Torix group. Nat Commun.

[16] Werren JH, Hurst GD, Zhang W, Breeuwer JA, Stouthamer R (1994). Rickettsial relative associated with male killing in the ladybird beetle (Adalia bipunctata). J Bacteriol.

[17] Hagimori T, Abe Y, Date S, Miura K (2006). The first finding of a Rickettsia bacterium associated with parthenogenesis induction among insects. Curr Microbiol.

[18] Łukasik P, van Asch M, Guo H, Ferrari J, Godfray HCJ (2013). Unrelated facultative endosymbionts protect aphids against a fungal pathogen. Ecol Lett.

[19] Hendry TA, Hunter MS, Baltrus DA (2014). The Facultative Symbiont Rickettsia Protects an Invasive Whitefly against Entomopathogenic Pseudomonas syringae Strains. Appl Environ Microbiol.

[20] Kliot A, Cilia M, Czosnek H, Ghanim M (2014). Implication of the bacterial endosymbiont Rickettsia spp. in interactions of the whitefly Bemisia tabaci with tomato yellow leaf curl virus. J Virol.

[21] Vanthournout B, Hendrickx F (2015). Endosymbiont dominated bacterial communities in a dwarf spider. PLoS One.

[22] Köstlbacher S, Collingro A, Halter T, Schulz F, Jungbluth SP (2021). Pangenomics reveals alternative environmental lifestyles among chlamydiae. Nat Commun.

[23] Kostanjšek R, Štrus J, Drobne D, Avguštin G (2004). “Candidatus Rhabdochlamydia porcellionis”, an intracellular bacterium from the hepatopancreas of the terrestrial isopod Porcellio scaber (Crustacea: Isopoda). Int J Syst Evol Microbiol.

[24] Corsaro D, Thomas V, Goy G, Venditti D, Radek R (2007). “Candidatus Rhabdochlamydia crassificans”, an intracellular bacterial pathogen of the cockroach Blatta orientalis (Insecta: Blattodea). Syst Appl Microbiol.

[25] Pillonel T, Bertelli C, Aeby S, de Barsy M, Jacquier N (2019). Sequencing the Obligate Intracellular Rhabdochlamydia helvetica within Its Tick Host Ixodes ricinus to Investigate Their Symbiotic Relationship. Genome Biol Evol.

[26] Radek R (2000). Light and electron microscopic study of a Rickettsiella species from the cockroach Blatta orientalis. J Invertebr Pathol.

[27] Kostanjšek R, Pirc Marolt T (2015). Pathogenesis, tissue distribution and host response to Rhabdochlamydia porcellionis infection in rough woodlouse Porcellio scaber. J Invertebr Pathol.

[28] Goodacre SL, Martin OY, Thomas CFG, Hewitt GM (2006). Wolbachia and other endosymbiont infections in spiders. Mol Ecol.

[29] Zhang L, Yun Y, Hu G, Peng Y (2018). Insights into the bacterial symbiont diversity in spiders. Ecol Evol.

[30] Sheffer MM, Uhl G, Prost S, Lueders T, Urich T (2019). Tissue- and Population-Level Microbiome Analysis of the Wasp Spider *Argiope bruennichi* Identified a Novel Dominant Bacterial Symbiont. Microorganisms.

[31] Rosenwald LC, Sitvarin MI, White JA (2020). Endosymbiotic *Rickettsiella* causes cytoplasmic incompatibility in a spider host. Proc Biol Sci.

[32] White JA, Styer A, Rosenwald LC, Curry MM, Welch KD (2020). Endosymbiotic Bacteria Are Prevalent and Diverse in Agricultural Spiders. Microb Ecol.

[33] Wernegreen JJ (2002). Genome evolution in bacterial endosymbionts of insects. Nat Rev Genet.

[34] Gil R, Latorre A, Moya A (2004). Bacterial endosymbionts of insects: insights from comparative genomics. Environ Microbiol.

[35] Moran NA (2007). Symbiosis as an adaptive process and source of phenotypic complexity. Proc Natl Acad Sci U S A.

[36] Sloan DB, Moran NA (2013). The evolution of genomic instability in the obligate endosymbionts of whiteflies. Genome Biol Evol.

[37] Hendrickx F, De Corte Z, Sonet G, Van Belleghem SM, Köstlbacher S (2022). A masculinizing supergene underlies an exaggerated male reproductive morph in A spider. Nat Ecol Evol.

[38] Li H (2018). Minimap2: pairwise alignment for nucleotide sequences. Bioinformatics.

[39] Wick RR, Judd LM, Gorrie CL, Holt KE (2017). Unicycler: Resolving bacterial genome assemblies from short and long sequencing reads. PLoS Comput Biol.

[40] Parks DH, Imelfort M, Skennerton CT, Hugenholtz P, Tyson GW (2015). CheckM: assessing the quality of microbial genomes recovered from isolates, single cells, and metagenomes. Genome Res.

[41] Wick RR, Schultz MB, Zobel J, Holt KE (2015). Bandage: interactive visualization of de novo genome assemblies. Bioinformatics.

[42] Li D, Liu C-M, Luo R, Sadakane K, Lam T-W (2015). MEGAHIT: an ultra-fast single-node solution for large and complex metagenomics assembly via succinct de Bruijn graph. Bioinformatics.

[43] Kang DD, Li F, Kirton E, Thomas A, Egan R (2019). MetaBAT 2: an adaptive binning algorithm for robust and efficient genome reconstruction from metagenome assemblies. PeerJ.

[44] Langmead B, Salzberg SL (2012). Fast gapped-read alignment with Bowtie 2. Nat Methods.

[45] Worning P, Jensen LJ, Hallin PF, Staerfeldt H-H, Ussery DW (2006). Origin of replication in circular prokaryotic chromosomes. Environ Microbiol.

[46] Gao F, Zhang C-T (2008). Ori-Finder: A web-based system for finding oriC s in unannotated bacterial genomes. BMC Bioinformatics.

[47] Ioannidis P, Hotopp JCD, Sapountzis P, Siozios S, Tsiamis G (2007). New criteria for selecting the origin of DNA replication in Wolbachia and closely related bacteria. BMC Genomics.

[48] Schmieder R, Edwards R (2011). Quality control and preprocessing of metagenomic datasets. Bioinformatics.

[49] Danecek P, Bonfield JK, Liddle J, Marshall J, Ohan V (2021). Twelve years of SAMtools and BCFtools. Gigascience.

[50] Katoh K, Standley DM (2013). MAFFT Multiple Sequence Alignment Software Version 7: Improvements in Performance and Usability. Molecular Biology and Evolution.

[51] Talavera G, Castresana J (2007). Improvement of phylogenies after removing divergent and ambiguously aligned blocks from protein sequence alignments. Syst Biol.

[52] Ronquist F, Teslenko M, van der Mark P, Ayres DL, Darling A (2012). MrBayes 3.2: efficient Bayesian phylogenetic inference and model choice across a large model space. Syst Biol.

[53] Edgar RC (2004). MUSCLE: multiple sequence alignment with high accuracy and high throughput. Nucleic Acids Res.

[54] Borowiec ML (2016). AMAS: a fast tool for alignment manipulation and computing of summary statistics. PeerJ.

[55] Minh BQ, Schmidt HA, Chernomor O, Schrempf D, Woodhams MD (2020). IQ-TREE 2: New Models and Efficient Methods for Phylogenetic Inference in the Genomic Era. Mol Biol Evol.

[56] Konstantinidis KT, Tiedje JM (2005). Towards a genome-based taxonomy for prokaryotes. J Bacteriol.

[57] Seemann T (2014). Prokka: rapid prokaryotic genome annotation. Bioinformatics.

[58] Nawrocki EP, Eddy SR (2013). Infernal 1.1: 100-fold faster RNA homology searches. Bioinformatics.

[59] Blin K, Shaw S, Kloosterman AM, Charlop-Powers Z, van Wezel GP (2021). antiSMASH 6.0: improving cluster detection and comparison capabilities. Nucleic Acids Res.

[60] Huerta-Cepas J, Szklarczyk D, Heller D, Hernández-Plaza A, Forslund SK (2019). eggNOG 5.0: a hierarchical, functionally and phylogenetically annotated orthology resource based on 5090 organisms and 2502 viruses. Nucleic Acids Res.

[61] Cantalapiedra CP, Hernández-Plaza A, Letunic I, Bork P, Huerta-Cepas J (2021). eggNOG-mapper v2: Functional Annotation, Orthology Assignments, and Domain Prediction at the Metagenomic Scale. Mol Biol Evol.

[62] Huerta-Cepas J, Szklarczyk D, Forslund K, Cook H, Heller D (2016). eggNOG 4.5: a hierarchical orthology framework with improved functional annotations for eukaryotic, prokaryotic and viral sequences. Nucleic Acids Res.

[63] Huerta-Cepas J, Forslund K, Coelho LP, Szklarczyk D, Jensen LJ (2017). Fast Genome-Wide Functional Annotation through Orthology Assignment by eggNOG-Mapper. Mol Biol Evol.

[64] Camacho C, Coulouris G, Avagyan V, Ma N, Papadopoulos J (2009). BLAST+: architecture and applications. BMC Bioinformatics.

[65] Miele V, Penel S, Duret L (2011). Ultra-fast sequence clustering from similarity networks with SiLiX. BMC Bioinformatics.

[66] Kurtz S, Phillippy A, Delcher AL, Smoot M, Shumway M (2004). Versatile and open software for comparing large genomes. Genome Biol.

[67] Krzywinski M, Schein J, Birol I, Connors J, Gascoyne R (2009). Circos: an information aesthetic for comparative genomics. Genome Res.

[68] R Core Team (2020). R: a language and environment for statistical computing. https://www.R-project.org/.

[69] Guy L, Kultima JR, Andersson SGE (2010). genoPlotR: comparative gene and genome visualization in R. Bioinformatics.

[70] Jones P, Binns D, Chang H-Y, Fraser M, Li W (2014). InterProScan 5: genome-scale protein function classification. Bioinformatics.

[71] Kanehisa M, Sato Y, Morishima K (2016). BlastKOALA and GhostKOALA: KEGG Tools for Functional Characterization of Genome and Metagenome Sequences. J Mol Biol.

[72] Kanehisa M, Sato Y, Kawashima M (2022). KEGG mapping tools for uncovering hidden features in biological data. Protein Sci.

[73] Siguier P, Perochon J, Lestrade L, Mahillon J, Chandler M (2006). ISfinder: the reference centre for bacterial insertion sequences. Nucleic Acids Res.

[74] Criscuolo A, Gribaldo S (2010). BMGE (Block Mapping and Gathering with Entropy): a new software for selection of phylogenetic informative regions from multiple sequence alignments. BMC Evol Biol.

[75] Zhu Q, Kosoy M, Dittmar K (2014). HGTector: an automated method facilitating genome-wide discovery of putative horizontal gene transfers. BMC Genomics.

[76] Deatherage DE, Barrick JE (2014). Identification of mutations in laboratory-evolved microbes from next-generation sequencing data using breseq. Methods Mol Biol.

[77] Wickham H (2016). Elegant Graphics for Data Analysis.

[78] Vanthournout B, Swaegers J, Hendrickx F (2011). Spiders do not escape reproductive manipulations by Wolbachia. BMC Evol Biol.

[79] Vanthournout B, Hendrickx F (2016). Hidden suppression of sex ratio distortion suggests Red queen dynamics between Wolbachia and its dwarf spider host. J Evol Biol.

[80] Halter T, Köstlbacher S, Collingro A, Sixt BS, Tönshoff ER (2022). Ecology and evolution of chlamydial symbionts of arthropods. ISME Commun.

[81] Bowers RM, Kyrpides NC, Stepanauskas R, Harmon-Smith M, Doud D (2017). Minimum information about a single amplified genome (MISAG) and a metagenome-assembled genome (MIMAG) of bacteria and archaea. Nat Biotechnol.

[82] Borges V, Nunes A, Ferreira R, Borrego MJ, Gomes JP (2012). Directional evolution of Chlamydia trachomatis towards niche-specific adaptation. J Bacteriol.

[83] Joseph SJ, Didelot X, Rothschild J, de Vries HJC, Morré SA (2012). Population genomics of Chlamydia trachomatis: insights on drift, selection, recombination, and population structure. Mol Biol Evol.

[84] Abdelsamed H, Peters J, Byrne GI (2013). Genetic variation in Chlamydia trachomatis and their hosts: impact on disease severity and tissue tropism. Future Microbiol.

[85] Nakamura Y, Kawai S, Yukuhiro F, Ito S, Gotoh T (2009). Prevalence of Cardinium bacteria in planthoppers and spider mites and taxonomic revision of “Candidatus Cardinium hertigii” based on detection of a new Cardinium group from biting midges. Appl Environ Microbiol.

[86] Edlund A, Ek K, Breitholtz M, Gorokhova E (2012). Antibiotic-induced change of bacterial communities associated with the copepod Nitocra spinipes. PLoS One.

[87] Santos-Garcia D, Rollat-Farnier P-A, Beitia F, Zchori-Fein E, Vavre F (2014). The genome of Cardinium cBtQ1 provides insights into genome reduction, symbiont motility, and its settlement in Bemisia tabaci. Genome Biol Evol.

[88] Erban T, Klimov P, Molva V, Hubert J (2020). Whole genomic sequencing and sex-dependent abundance estimation of Cardinium sp., a common and hyperabundant bacterial endosymbiont of the American house dust mite, Dermatophagoides farinae. Exp Appl Acarol.

[89] Moffitt MC, Neilan BA (2000). The expansion of mechanistic and organismic diversity associated with non-ribosomal peptides. FEMS Microbiol Lett.

[90] Pilgrim J, Ander M, Garros C, Baylis M, Hurst GDD (2017). Torix group Rickettsia are widespread in Culicoides biting midges (Diptera: Ceratopogonidae), reach high frequency and carry unique genomic features. Environ Microbiol.

[91] Bordenstein SR, Reznikoff WS (2005). Mobile DNA in obligate intracellular bacteria. Nat Rev Microbiol.

[92] Moran NA, Plague GR (2004). Genomic changes following host restriction in bacteria. Curr Opin Genet Dev.

[93] Neupane S, Bonilla SI, Manalo AM, Pelz-Stelinski KS (2022). Complete de novo assembly of Wolbachia endosymbiont of Diaphorina citri Kuwayama (Hemiptera: Liviidae) using long-read genome sequencing. Sci Rep.

[94] Perlmutter JI, Bordenstein SR, Unckless RL, LePage DP, Metcalf JA (2019). The phage gene wmk is a candidate for male killing by a bacterial endosymbiont. PLoS Pathog.

[95] Perlmutter JI, Meyers JE, Bordenstein SR (2021). A single synonymous nucleotide change impacts the male-killing phenotype of prophage WO gene wmk. Elife.

[96] Nikoh N, Hosokawa T, Moriyama M, Oshima K, Hattori M (2014). Evolutionary origin of insect-Wolbachia nutritional mutualism. Proc Natl Acad Sci U S A.

[97] Gerth M, Bleidorn C (2016). Comparative genomics provides a timeframe for Wolbachia evolution and exposes a recent biotin synthesis operon transfer. Nat Microbiol.

[98] Ju J-F, Bing X-L, Zhao D-S, Guo Y, Xi Z (2020). Wolbachia supplement biotin and riboflavin to enhance reproduction in planthoppers. ISME J.

[99] Driscoll TP, Verhoeve VI, Brockway C, Shrewsberry DL, Plumer M (2020). Evolution of *Wolbachia* mutualism and reproductive parasitism: insight from two novel strains that co-infect cat fleas. PeerJ.

[100] Costa TRD, Felisberto-Rodrigues C, Meir A, Prevost MS, Redzej A (2015). Secretion systems in Gram-negative bacteria: structural and mechanistic insights. Nat Rev Microbiol.

[101] Böck D, Medeiros JM, Tsao H-F, Penz T, Weiss GL (2017). In situ architecture, function, and evolution of a contractile injection system. Science.

[102] Van Melderen L (2010). Toxin-antitoxin systems: why so many, what for?. Curr Opin Microbiol.

[103] Unterholzner SJ, Poppenberger B, Rozhon W (2013). Toxin-antitoxin systems: Biology, identification, and application. Mob Genet Elements.

[104] Fraikin N, Goormaghtigh F, Van Melderen L (2020). Type II Toxin-Antitoxin Systems: Evolution and Revolutions. J Bacteriol.

[105] Massey JH, Newton ILG (2022). Diversity and function of arthropod endosymbiont toxins. Trends Microbiol.

[106] Singhal K, Mohanty S (2018). Comparative genomics reveals the presence of putative toxin–antitoxin system in Wolbachia genomes. Mol Genet Genomics.

[107] Ferrell JC, Fields KA (2016). A working model for the type III secretion mechanism in Chlamydia. Microbes Infect.

[108] Mann E, Stouthamer CM, Kelly SE, Dzieciol M, Hunter MS Transcriptome sequencing reveals novel candidate genes for cardinium hertigii-caused cytoplasmic incompatibility and host-cell interaction. msystems;2. epub ahead of print november 2017.

[109] Gillespie JJ, Ammerman NC, Dreher-Lesnick SM, Rahman MS, Worley MJ (2009). An Anomalous Type IV Secretion System in Rickettsia Is Evolutionarily Conserved. PLoS ONE.

[110] Pichon S, Bouchon D, Cordaux R, Chen L, Garrett RA (2009). Conservation of the Type IV Secretion System throughout Wolbachia evolution. Biochemical and Biophysical Research Communications.

[111] Ogata H, La Scola B, Audic S, Renesto P, Blanc G (2006). Genome sequence of Rickettsia bellii illuminates the role of amoebae in gene exchanges between intracellular pathogens. PLoS Genet.

[112] Gillespie JJ, Joardar V, Williams KP, Driscoll T, Hostetler JB (2012). A Rickettsia genome overrun by mobile genetic elements provides insight into the acquisition of genes characteristic of an obligate intracellular lifestyle. J Bacteriol.

[113] Gillespie JJ, Kaur SJ, Rahman MS, Rennoll-Bankert K, Sears KT (2015). Secretome of obligate intracellular Rickettsia. FEMS Microbiol Rev.

[114] Hagen R, Verhoeve VI, Gillespie JJ, Driscoll TP (2018). Conjugative Transposons and Their Cargo Genes Vary across Natural Populations of Rickettsia buchneri Infecting the Tick Ixodes scapularis. Genome Biol Evol.

[115] Martyn JE, Gomez-Valero L, Buchrieser C (2022). The evolution and role of eukaryotic-like domains in environmental intracellular bacteria: the battle with a eukaryotic cell. FEMS Microbiol Rev.

[116] Frank AC (2019). Molecular host mimicry and manipulation in bacterial symbionts. FEMS Microbiol Lett.

[117] Zeng Z, Fu Y, Guo D, Wu Y, Ajayi OE (2018). Bacterial endosymbiont Cardinium cSfur genome sequence provides insights for understanding the symbiotic relationship in Sogatella furcifera host. BMC Genomics.

[118] Siozios S, Ioannidis P, Klasson L, Andersson SGE, Braig HR (2013). The diversity and evolution of Wolbachia ankyrin repeat domain genes. PLoS One.

[119] Sinha A, Li Z, Sun L, Carlow CKS (2019). Complete Genome Sequence of the Wolbachia wAlbB Endosymbiont of Aedes albopictus. Genome Biol Evol.

[120] Siguier P, Filée J, Chandler M (2006). Insertion sequences in prokaryotic genomes. Curr Opin Microbiol.

[121] Touchon M, Rocha EPC (2007). Causes of insertion sequences abundance in prokaryotic genomes. Mol Biol Evol.

[122] Latorre A, Manzano-Marín A (2017). Dissecting genome reduction and trait loss in insect endosymbionts. Ann N Y Acad Sci.

[123] Plague GR, Dunbar HE, Tran PL, Moran NA (2008). Extensive proliferation of transposable elements in heritable bacterial symbionts. J Bacteriol.

[124] Newton ILG, Bordenstein SR (2011). Correlations between bacterial ecology and mobile DNA. Curr Microbiol.

[125] Ros VID, Fleming VM, Feil EJ, Breeuwer JAJ (2012). Diversity and recombination in Wolbachia and Cardinium from Bryobia spider mites. BMC Microbiol.

[126] Le Clec’h W, Chevalier FD, Genty L, Bertaux J, Bouchon D (2013). Cannibalism and predation as paths for horizontal passage of Wolbachia between terrestrial isopods. PLoS One.

[127] Gonella E, Pajoro M, Marzorati M, Crotti E, Mandrioli M (2015). Plant-mediated interspecific horizontal transmission of an intracellular symbiont in insects. Sci Rep.

[128] Li S-J, Ahmed MZ, Lv N, Shi P-Q, Wang X-M (2017). Plantmediated horizontal transmission of Wolbachia between whiteflies. ISME J.

[129] Li Y-H, Ahmed MZ, Li S-J, Lv N, Shi P-Q (2017). Plant-mediated horizontal transmission of Rickettsia endosymbiont between different whitefly species. FEMS Microbiol Ecol.

[130] Turelli M, Cooper BS, Richardson KM, Ginsberg PS, Peckenpaugh B (2018). Rapid Global Spread of wRi-like Wolbachia across Multiple Drosophila. Curr Biol.

[131] Stouthamer CM, Kelly SE, Mann E, Schmitz-Esser S, Hunter MS (2019). Development of a multi-locus sequence typing system helps reveal the evolution of Cardinium hertigii, a reproductive manipulator symbiont of insects. BMC Microbiol.

[132] Park E, Poulin R (2020). Widespread Torix Rickettsia in New Zealand amphipods and the use of blocking primers to rescue host COI sequences. Sci Rep.

[133] El Karkouri K, Pontarotti P, Raoult D, Fournier P-E (2016). Origin and Evolution of Rickettsial Plasmids. PLoS One.

[134] El Karkouri K, Ghigo E, Raoult D, Fournier P-E (2022). Genomic evolution and adaptation of arthropod-associated *Rickettsia*. Sci Rep.

[135] Duron O (2013). Lateral transfers of insertion sequences between *Wolbachia, Cardinium and Rickettsia* bacterial endosymbionts. Heredity (Edinb).

