# Supplementary material for: One to host them all: genomics of the diverse bacterial endosymbionts of the spider Oedothorax gibbosus
Source: Microb Genom. 2023 Feb 9;9(2):mgen000943. doi: 10.1099/mgen.0.000943 (PMC9997750; doi:10.1099/mgen.0.000943)
Supplement: Supplementary material 1 [file mgen-9-943-s001.pdf]

Supplemetary Methods

'*Candidatus* Tisiphia' 16S phylogeny

To calculate the 16S rRNA gene phylogeny for the genus '*Candidatus* Tisiphia' we downloaded all available *Rickettsia* and '*Candidatus* Tisiphia' 16S rRNA gene sequences from SILVA SSU (r138.1) on the 16th November 2021. Further, we added 16S rRNA gene sequences from the dataset we used for the ribosomal protein gene phylogeny (**Table S8**) and sequences obtained from other arachnid hosts [1]. We then removed duplicates and filtered the sequences to remove sequences shorter than 1,000 bp. The remaining sequences were aligned using SINA ("variability profile: bacteria") [2] and trimmed using trimAl (v1.4.rev15; "-noallgaps") [3]. The phylogeny was then calculated with iqtree2 (v2.1.2; "-bnni" "-alrt 1000" "-m TESTNEW" "--madd LG4X" "-bb 1000") using the TVMe+R6 substitution model [4].

References

1. **Pilgrim J, Thongprem P, Davison HR, Siozios S, Baylis M, et al.** Torix *Rickettsia* are widespread in arthropods and reflect a neglected symbiosis. *Gigascience*;10. Epub ahead of print 25 March 2021. DOI: 10.1093/gigascience/giab021.
2. **Pruesse E, Peplies J, Glöckner FO.** SINA: accurate high-throughput multiple sequence alignment of ribosomal RNA genes. Bioinformatics 2012;28:1823–1829.
3. **Capella-Gutiérrez S, Silla-Martínez JM, Gabaldón T.** trimAl: a tool for automated alignment trimming in large-scale phylogenetic analyses. *Bioinformatics* 2009; 25:1972–1973.
4. **Minh BQ, Schmidt HA, Chernomor O, Schrempf D, Woodhams MD, et al.** IQ-TREE 2: New Models and Efficient Methods for Phylogenetic Inference in the Genomic Era. *Mol Biol Evol* 2020;37:1530–1534.

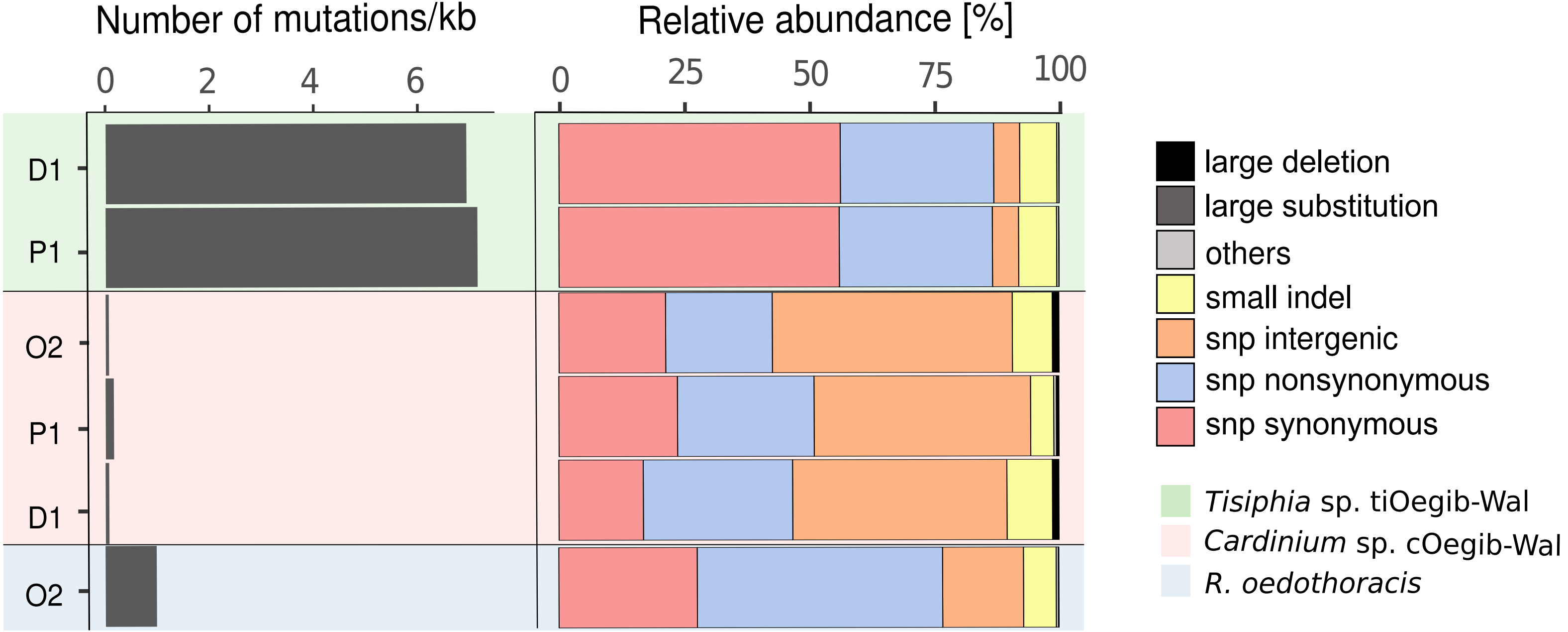

Figure S1. Frequency and relative abundance of genomic variations found in '*Candidatus* Tisiphia', *Cardinium* and *Rhabdochlamydia* endosymbionts of selected *O. gibbosus* populations.

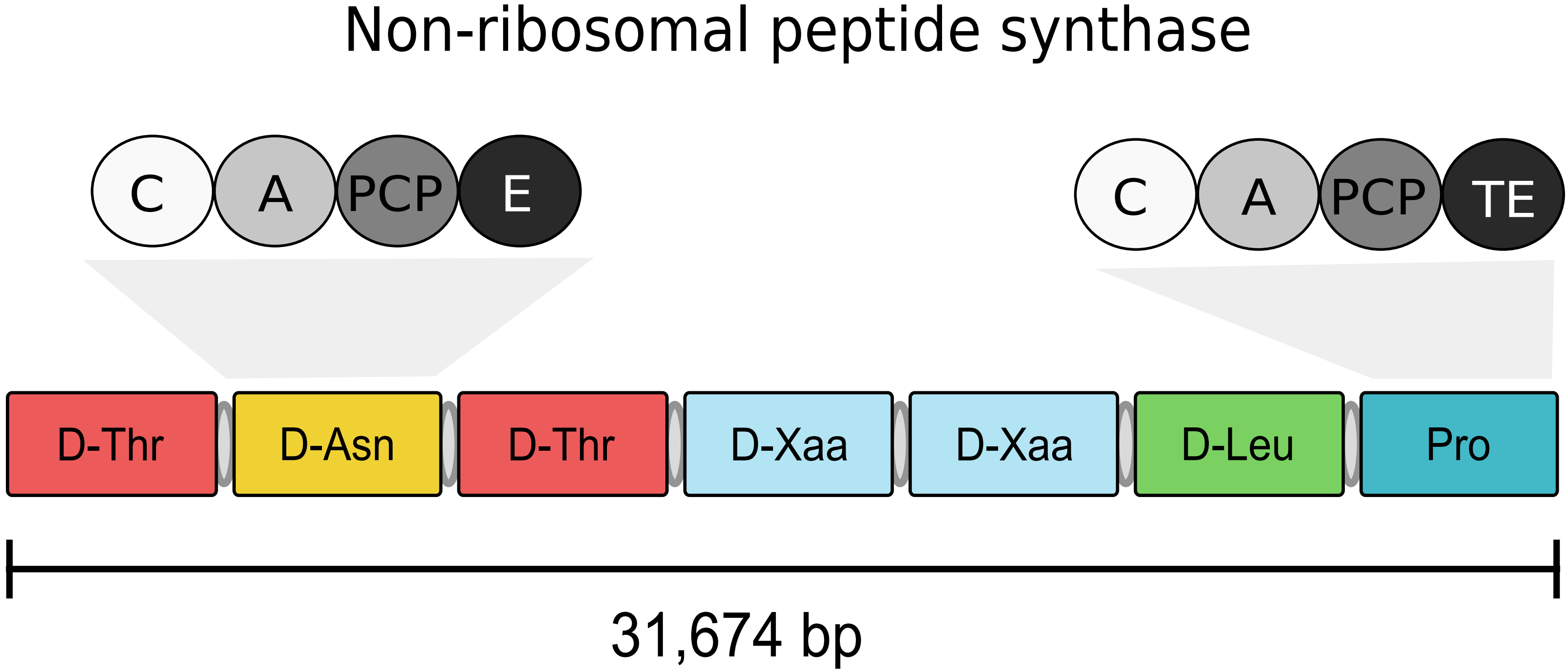

Figure S2. Structure of the non-ribosomal peptide synthase in the genome of *Cardinium* sp. cOegib-Wal. The NRPS consists of seven modules, each incorporating oneamino acid (Thr - Threonine, Asn - Asparagine, Leu - Leucine, Pro - Proline, Xaa - Any aminoacid). Each module consists of four domains: an adenylation domain (A), an peptidyl carrierprotein domain (PCP), a condensation domain (C) and an epimerization domain (E). The lastmodule contains a thioesterase domain (TE) instead of an epimerization domain.

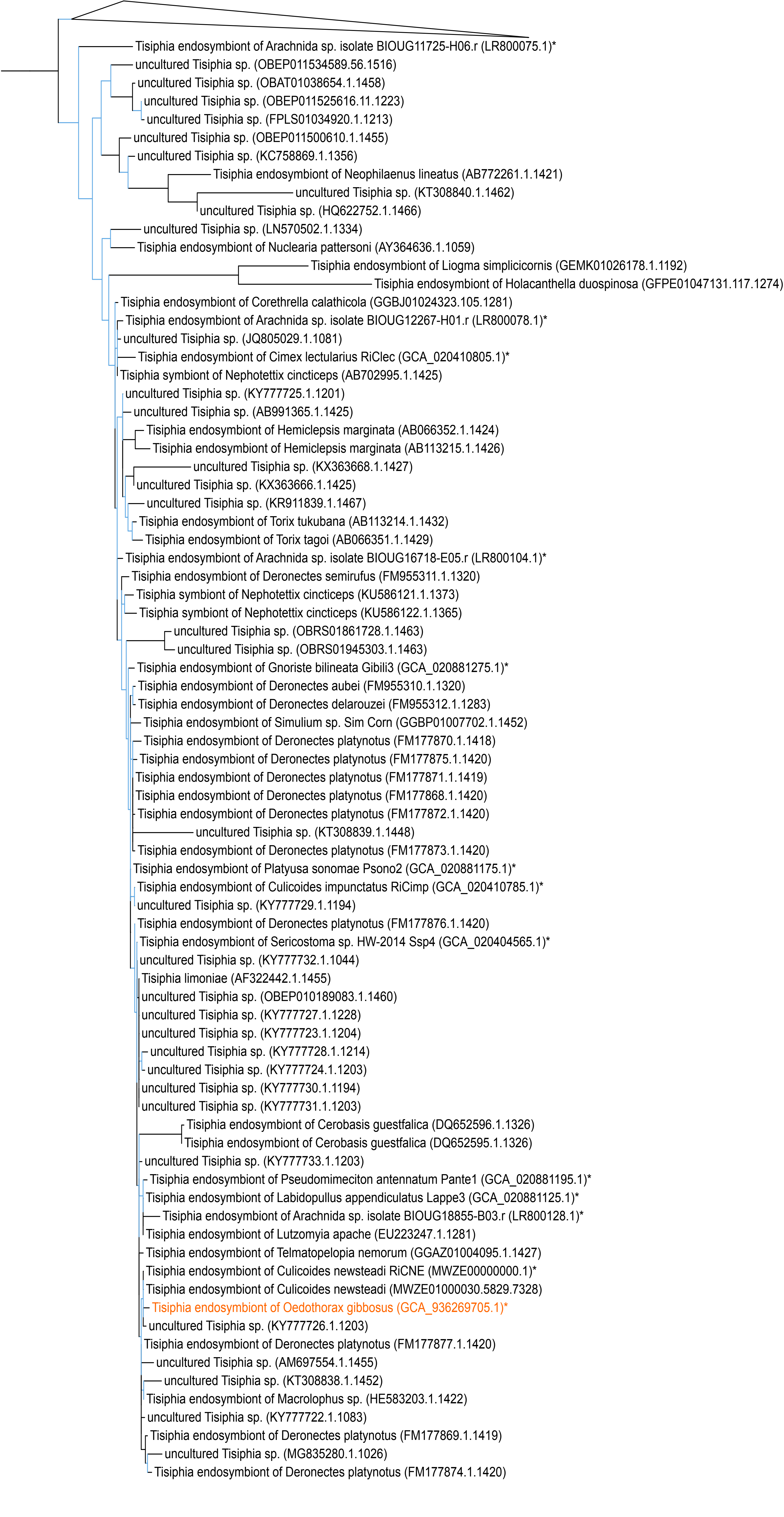

Figure S3. 16S rRNA gene phylogeny of the genus '*Candidatus* Tisiphia'. Scalebar indicates 0.1 substitutions per position in the alignment. The tree was rooted usingOrientia sp. and '*Candidatus* Megaira' sp. as an outgroup for visualization. Branches withbootstrap values >95 are indicated in black, branches with lower bootstrap values are shownin blue. For visualization sequences belonging to the genus *Rickettsia* were collapsed. The sequence of the *Tisiphia* endosymbiont of *O. gibbosus* is shown in orange. The SILVA accession numbers are written in parentheses. 16S rRNA sequences that were extractedfrom genome sequences are indicated by an asterisk and the respective NCBI accessionnumber is written in parentheses.

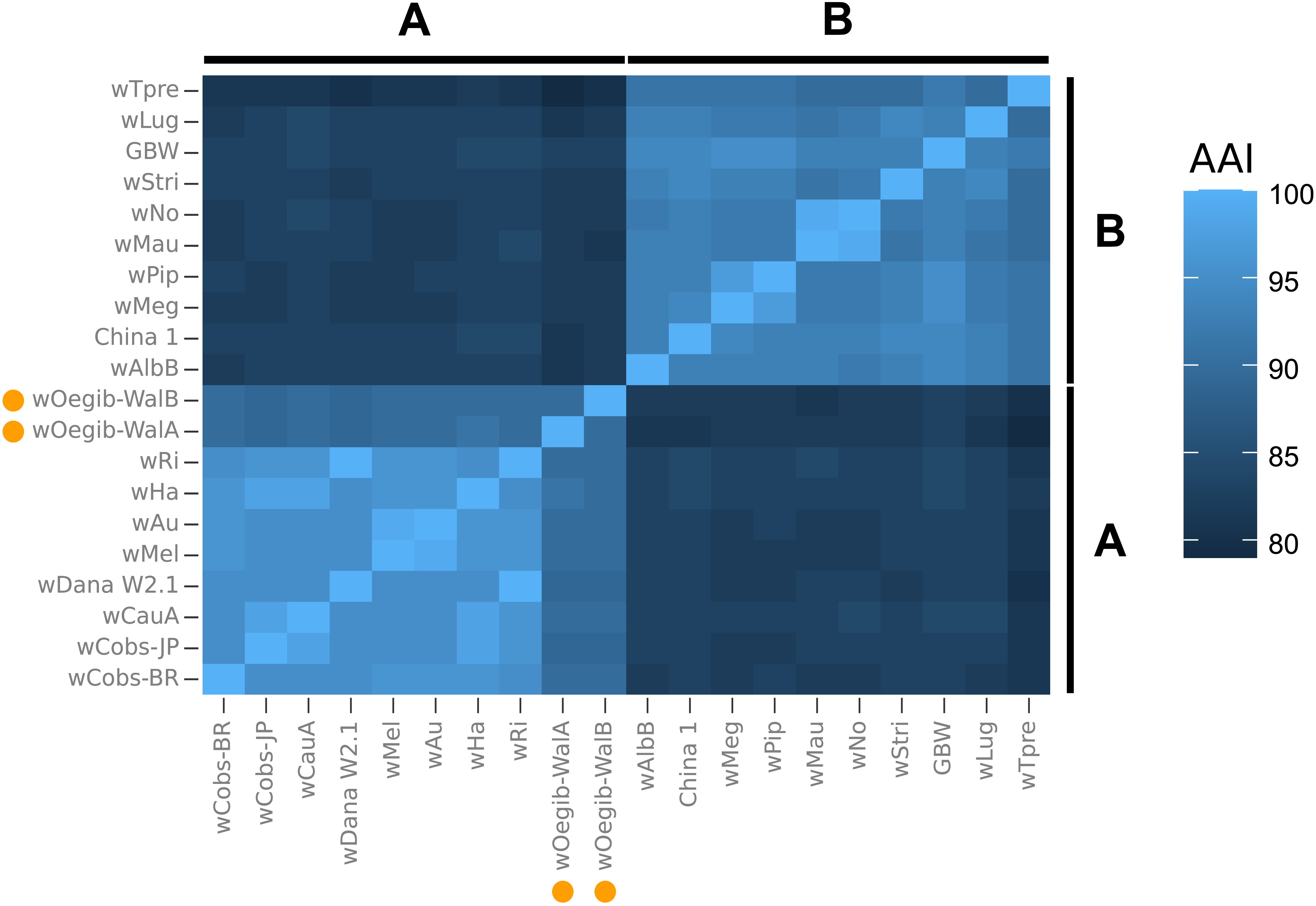

Figure S4. Average Amino Acid Identity (AAI) of different members ofsupergroup A and B of the genus *Wolbachia*. The AAI was calculated as described in Konstantinidis KT and Tiedje JM. *J Bacteriol* (2005) **187**:6258-6264 **doi:** 10.1128/jb 187.18.6258-6264.2005.

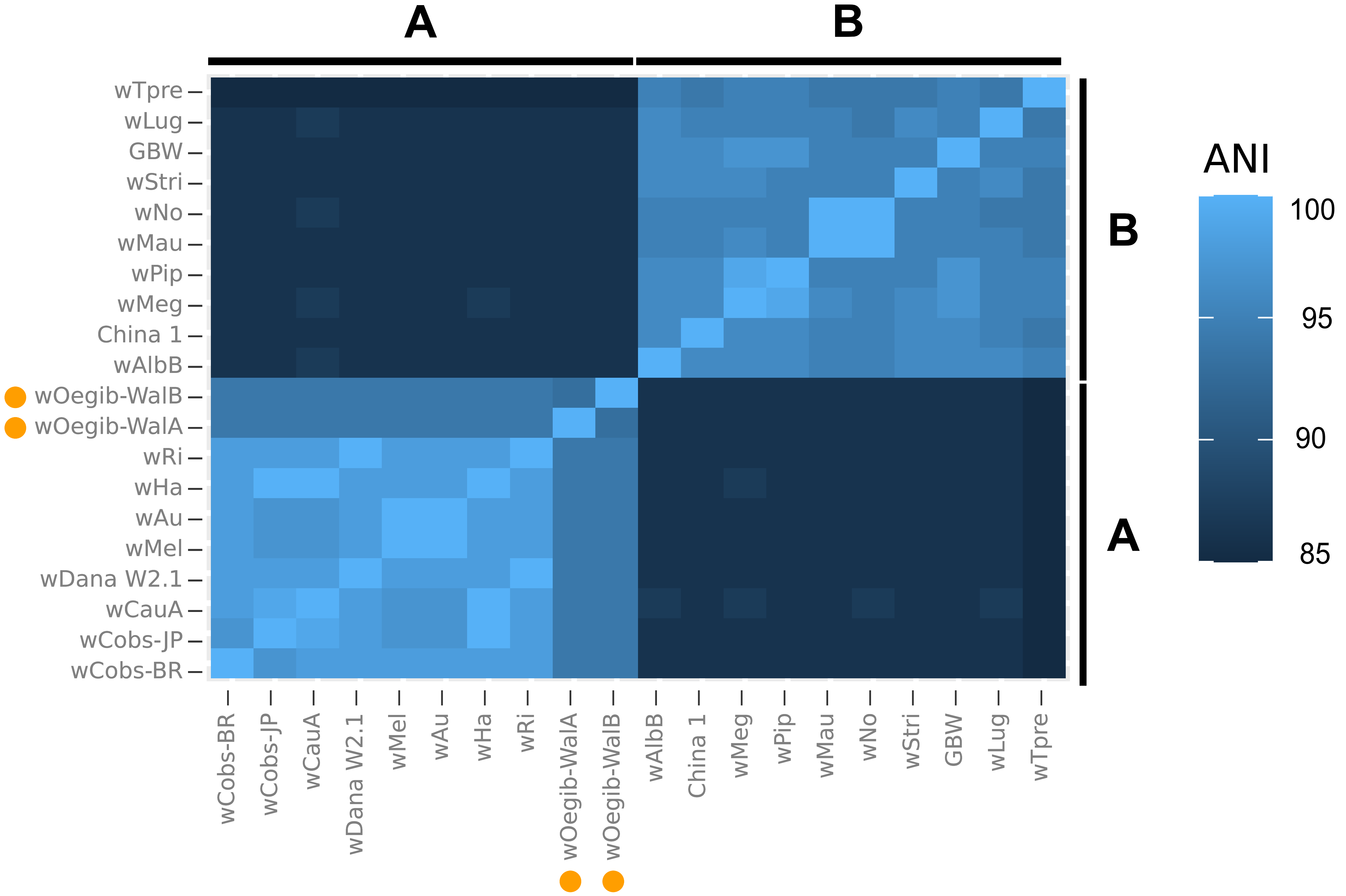

Figure S5. Average Nucleotide Identity (ANI) of different members of supergroup A and B of the genus *Wolbachia*. The ANI was calculated as described in Konstantinidis KT and Tiedje JM. *J Bacteriol* (2005) **187**:6258-6264 **doi:** 10.1128/jb 187.18.6258-6264.2005.

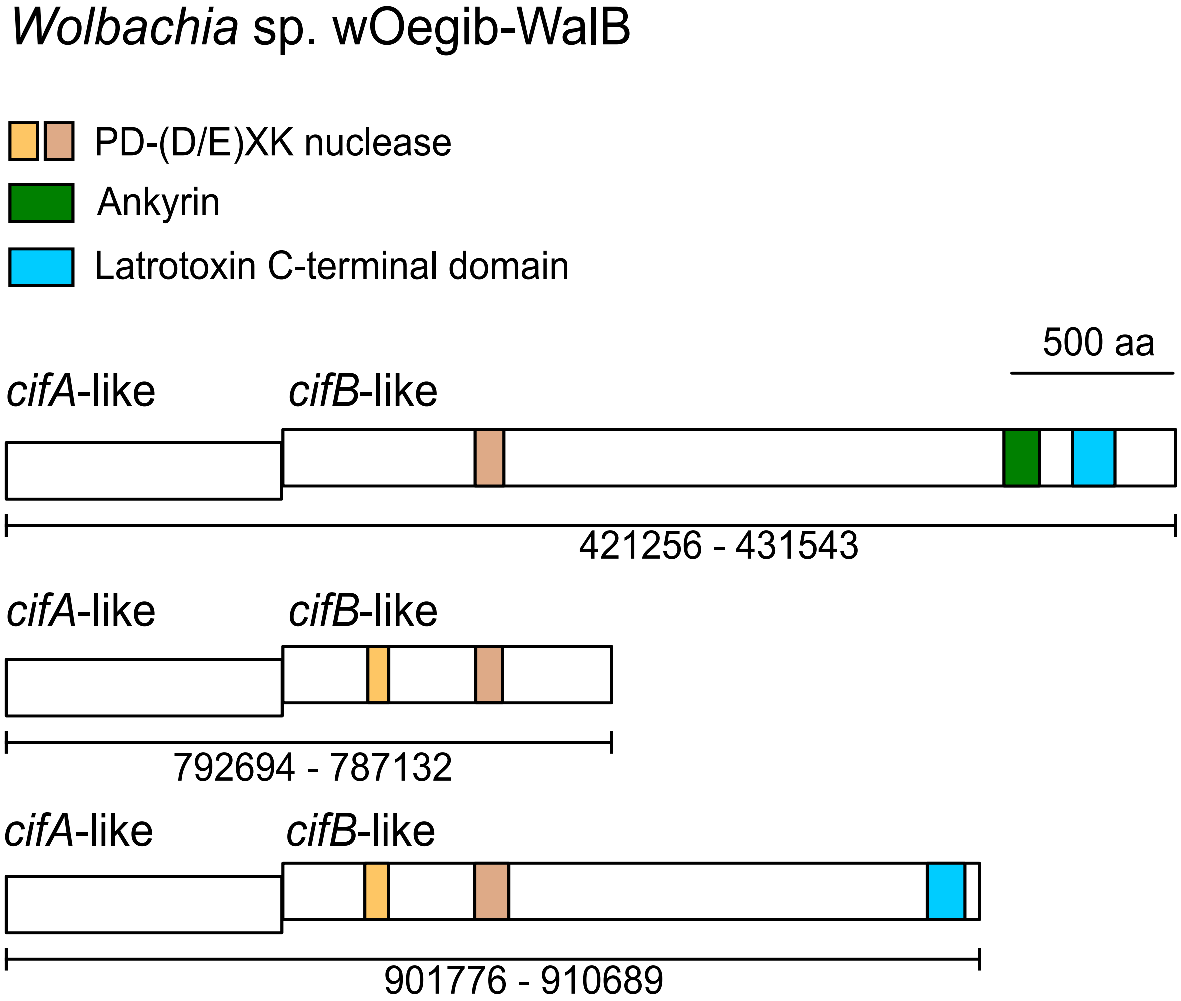

Figure S6. Analysis of putative *cif* gene clusters in *Wolbachia* sp. wOeGib-WalB. Remote homologs of *cifA*- and *cifB*-like genes potentially involved in cytoplasmic incompatibility in the genome of wOegib-WalB and identification of typical domains.

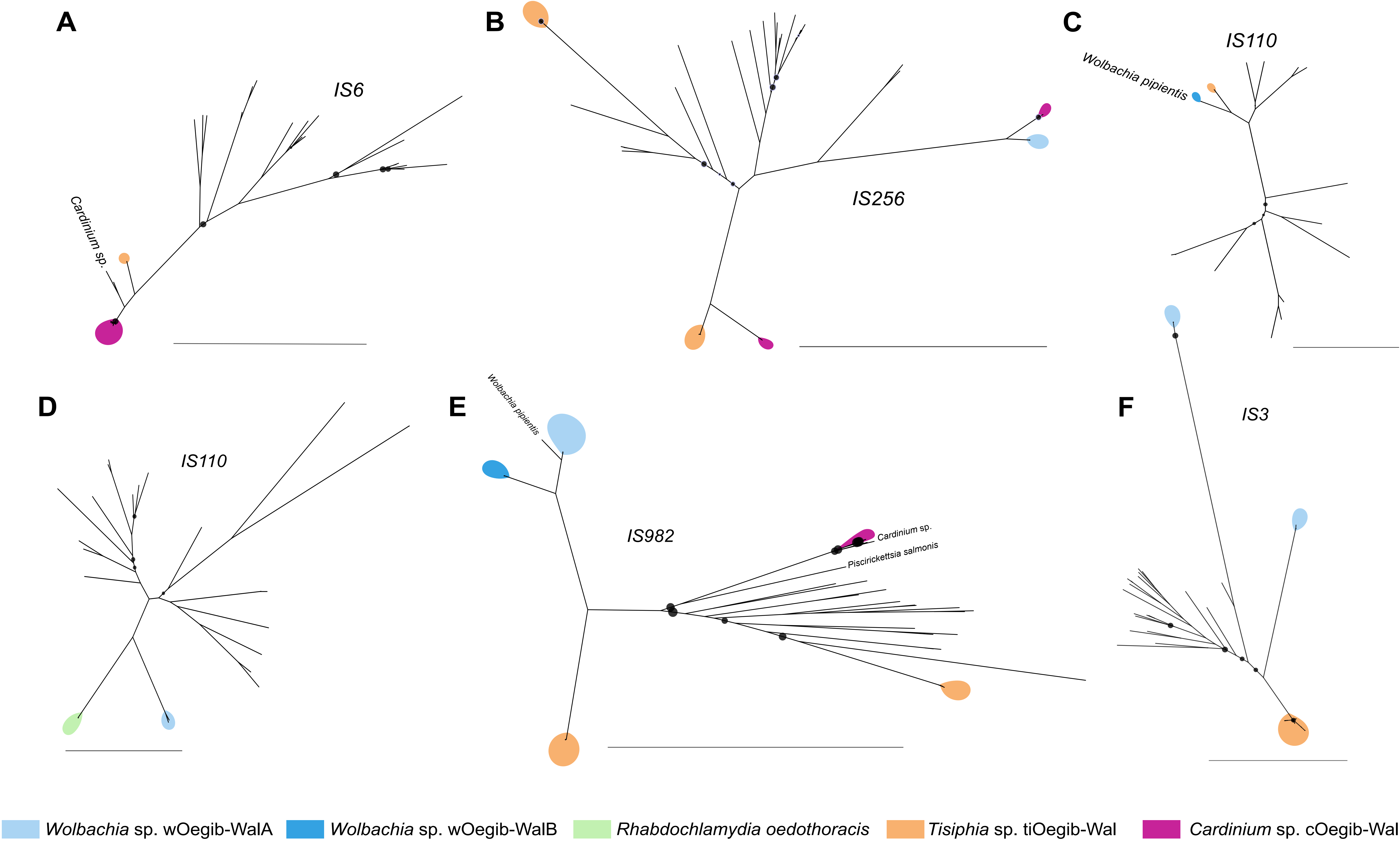

Figure S7. Phylogenies of de-novo clustered TE gene families. Scale bar indicates 0.1 substitutions per position in the alignment. Black dots indicate bootstrap values, where the size indicates values ranging from 0-95. Further details on the gene families can be found in **Table S7**. Note that for the trees only complete sequences were used, thus in some cases not all endosymbionts stated in **Table S7** are represented in the trees.
